# Transformer Models in Vibrational Spectroscopy: A Review

**DOI:** 10.3390/molecules31203745

**Published:** 2026-10-19

**Authors:** Jamile Mohammad Jafari, Thomas Bocklitz

**Affiliations:** 1 Leibniz Institute of Photonic Technology, Member of Leibniz Health Technologies, Member of the Leibniz Centre for Photonics in Infection Research (LPI), Albert-Einstein-Strasse 9, 07745 Jena, Germany; jamile.jafari@leibniz-ipht.de; 2 Institute of Physical Chemistry (IPC) and Abbe Center of Photonics (ACP), Friedrich Schiller University Jena, Member of the Leibniz Centre for Photonics in Infection Research (LPI), Helmholtzweg 4, 07743 Jena, Germany

**Keywords:** transformer model, deep learning, vibrational spectroscopy, chemometrics, machine learning

## Abstract

Transformer models have attracted increasing interest in vibrational spectroscopy because of their ability to capture long-range dependencies in spectral data. These models have been applied to analytical tasks including spectral preprocessing, classification, quantitative analysis, multimodal data fusion, and spectra-to-structure prediction. Their flexible architectures support applications in Raman, infrared (IR), near-infrared (NIR), and surface-enhanced Raman spectroscopy (SERS), with examples across chemistry, biology, and materials science. Transformer-based models also offer opportunities to integrate chemical, physical, and biological information in spectral analysis and support interdisciplinary research involving chemometrics, deep learning, and experimental spectroscopy. In this review, we elucidate the fundamental principles of Transformer models and their wide-ranging applications in vibrational spectroscopy, spanning from baseline correction and deconvolution to molecular identification, disease diagnosis, food authentication, and quantitative property prediction. Finally, as Transformer models may not be suitable for every analytical task or consistently outperform simpler approaches, we discuss current challenges and future trends in Transformer models in vibrational spectroscopy and related analytical sciences.

## 1. Introduction

**Figure 1:**
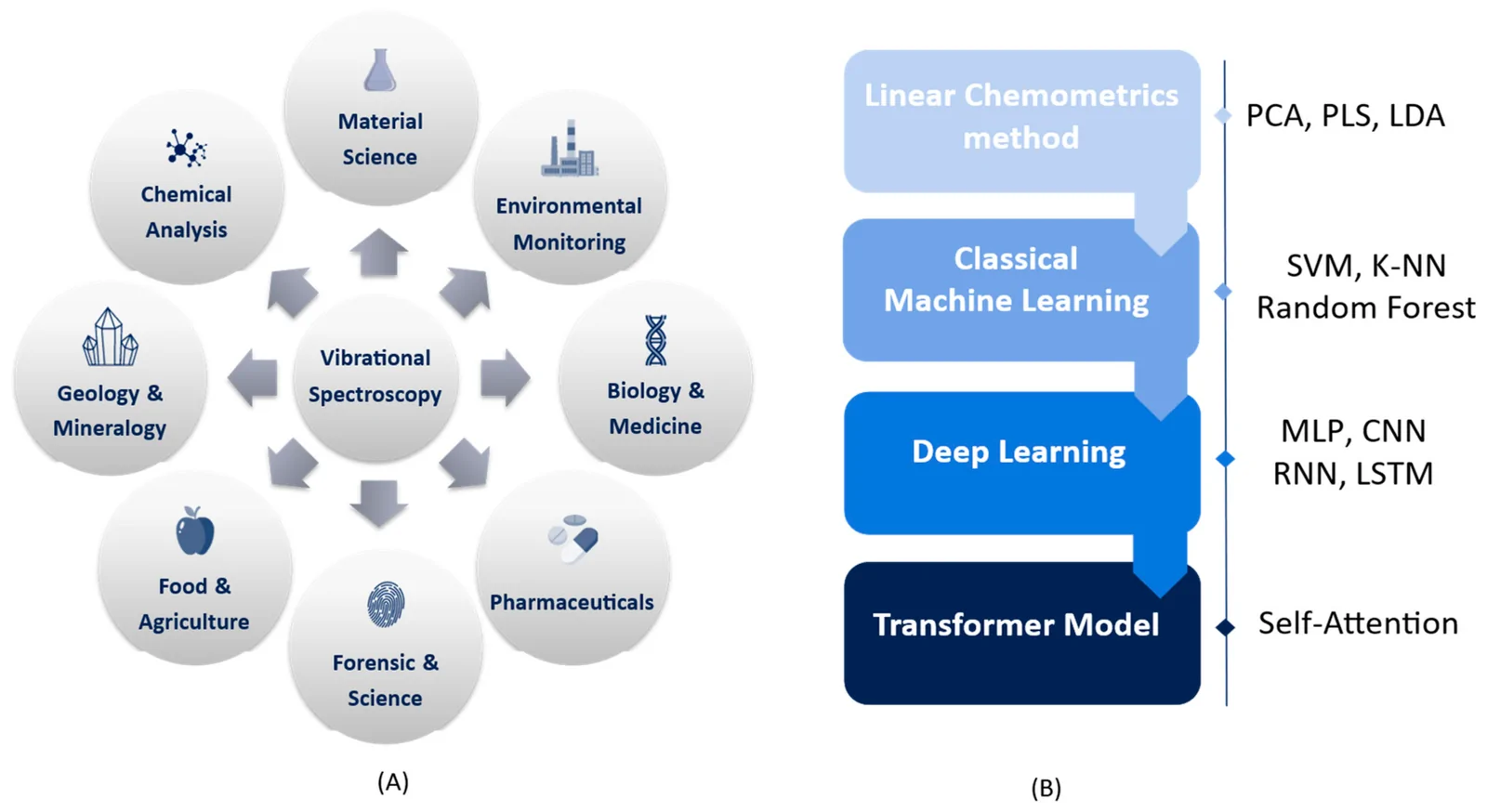
(**A**) Application of vibrational spectroscopy. (**B**) Evaluation of analysis methods in vibrational spectroscopy.

Vibrational spectroscopy is a fundamental analytical technique in chemistry, biology, and materials science. It examines quantized molecular vibrations, such as stretching, bending, and torsional modes, to provide chemically specific spectral fingerprints [1]. These vibrational transitions give rise to characteristic interactions with electromagnetic radiation at specific frequencies, enabling rapid, label-free, and non-destructive identification of chemical compounds [2]. Vibrational spectroscopy has become widely used across multiple fields (Figure 1A), including pharmaceuticals [3,4], biology and medicine [5,6,7,8], polymer and materials science [9,10], food and agriculture [11,12], forensic science [13], and environmental monitoring [14,15]. Its ability to provide detailed chemical information with minimal sample preparation makes it particularly suitable for high-throughput analysis. In addition, the rich information content of vibrational spectra provides an excellent foundation for computational and machine-learning models aimed at automated interpretation [16].

Traditional analysis of vibrational spectroscopy data has relied on classical chemometric and machine-learning methods [17,18]. These approaches enable dimensionality reduction [19], pattern recognition, and quantitative prediction. Principal component analysis (PCA) commonly extracts dominant variance and reduces spectral dimensionality [20]. Partial least squares (PLS) and its variants, such as PLS-DA, are widely applied for regression and classification tasks [21]. Linear discriminant analysis (LDA) remains a standard method for supervised classification [22], while support vector machines (SVMs) perform well in handling nonlinear decision boundaries in Raman and IR datasets [23]. Other machine-learning methods, including k-nearest neighbors (k-NN) and random forests (RF), have also demonstrated strong performance across a wide range of vibrational spectroscopy applications [24,25,26].

Despite their success, classical methods face limitations when applied to complex spectral data [24]. Many rely on linear assumptions, which can make them less effective at modeling nonlinear relationships arising from overlapping bands, fluorescence background signals, or environmental variability [27,28]. Vibrational spectroscopy data often exhibit overlapping peaks, baseline distortions, low signal-to-noise ratios, instrumental variability, and subtle spectral differences between classes, making robust automated analysis challenging. Classical approaches also depend heavily on preprocessing steps such as baseline correction, normalization, and smoothing, which introduce operator dependence and reduce reproducibility [29]. In addition, their performance often degrades when applied to data acquired from different instruments or under varying experimental conditions [30]. These limitations highlight the need for more flexible, data-driven approaches.

Deep-learning methods have been introduced to address these challenges [31,32]. Multi-layer perceptrons (MLPs) improve predictive performance in quantitative spectroscopy [33]. Convolutional neural networks (CNNs) effectively capture local spectral features and handle noise, while recurrent neural networks (RNNs), including long short-term memory (LSTM) models, are designed to capture sequential dependencies [34]. These approaches reduce the need for manual feature engineering and improve generalization across varying conditions.

However, conventional deep-learning models still have important limitations. In vibrational spectroscopy, diagnostically relevant information may be distributed across distant spectral regions, making purely local convolutional operations insufficient for capturing global spectral relationships. CNNs primarily focus on local patterns and may therefore have limited ability to capture long-range dependencies between distant spectral regions [35]. RNN-based models can capture longer dependencies but often suffer from vanishing gradients, sequential processing bottlenecks, and limited scalability. In addition, both approaches typically require large labeled datasets and remain sensitive to domain shifts and variations in experimental conditions [36].

To overcome these challenges, Transformer-based models have recently gained increasing attention [37]. Originally developed for natural language processing, Transformers process spectral sequences in parallel and efficiently capture long-range dependencies across the entire spectrum [38,39,40,41] (Figure 1B). This makes them particularly suitable for vibrational spectroscopy, where correlations between distant spectral regions can contain diagnostically or chemically relevant information. By leveraging attention mechanisms, Transformers can focus on the most informative spectral features while preserving global contextual relationships.

Although several reviews have discussed the application of machine-learning and deep-learning methods in vibrational spectroscopy [35,42], a focused examination of Transformer architectures across different spectroscopic techniques and analytical tasks offers a complementary perspective. Earlier reviews have surveyed deep-learning applications and provided practical guidance for developing convolutional neural network workflows [35], or discussed broader advances and limitations of deep learning, including transfer learning and data augmentation [42]. Building on these contributions, the present review focuses specifically on Transformer models in vibrational spectroscopy. We summarize their fundamental principles and discuss how they differ from traditional chemometric methods and conventional deep-learning approaches. We highlight recent applications in preprocessing, classification, regression, multimodal data fusion, and spectral interpretation across Raman, infrared, and near-infrared spectroscopy. By connecting Transformer architectures with these applications, this review provides a focused synthesis of their role in vibrational spectral analysis.

The literature for this review was identified through Google Scholar searches covering publications from 2017 to 2026. The search combined the terms “transformer”, “attention”, and “self-attention” with spectroscopic terms, including “Raman”, “infrared spectroscopy”, “near-infrared spectroscopy”, “Fourier transform infrared spectroscopy”, “vibrational spectroscopy”, and “spectroscopy”. The abbreviations “IR”, “NIR”, “FTIR”, and “SERS” were also used. Studies were screened for relevance to vibrational spectroscopy and included when the development or application of Transformer models was a central focus. The reference lists of relevant articles were also examined to identify additional studies.

## 2. Technical Basis of Transformer Models

**Figure 2:**
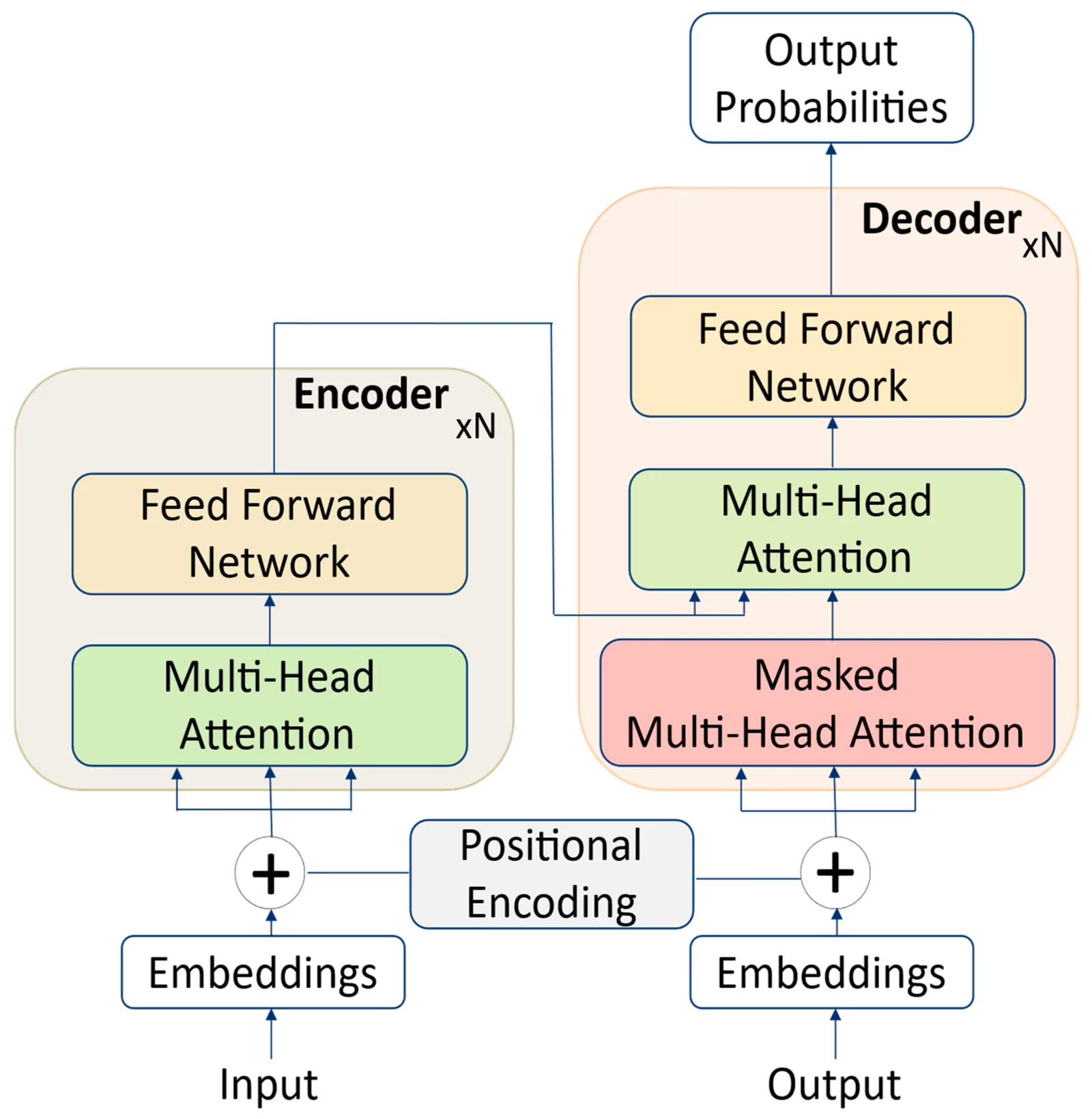
Schematic overview of the original Transformer encoder–decoder architecture. Based on the architecture described by Vaswani et al. [37].

The Transformer model proposed by Vaswani et al. [37] is a sequence-processing architecture based primarily on attention mechanisms. The original architecture comprises two main components, an encoder and a decoder, as illustrated in Figure 2. The encoder transforms the input sequence into contextualized representations, whereas the decoder uses the encoder output to generate the target sequence. Rather than relying on recurrent processing, the Transformer uses stacked attention and feedforward layers to model dependencies across the sequence while enabling parallel computation during training. The encoder consists of multiple structurally identical layers. Each encoder layer contains a multi-head self-attention module followed by a position-wise feedforward neural network. The self-attention module lets every element of the input sequence interact with the others, while the feedforward network applies nonlinear transformations to the representation generated at each position. Residual connections surround both components to facilitate information propagation through the network [43]. Layer normalization also improves training stability [44]. Together, these components allow the encoder to generate a contextualized representation of the complete input sequence.

The decoder has a structure similar to that of the encoder but includes an additional attention operation. First, it applies masked self-attention to the target sequence. The masking operation prevents each position from accessing subsequent target positions, thereby preserving the autoregressive nature of sequence generation. The decoder then applies cross-attention, where the decoder representations attend to the encoder output. A position-wise feedforward network subsequently transforms the resulting features. As in the encoder, each decoder sublayer is surrounded by a residual connection, followed by layer normalization [37].

Self-attention is the central operation of the Transformer. For each input element, learned linear projections generate three representations: the query, key, and value. Attention scores are obtained by comparing the queries with the keys. These scores are scaled and normalized, then used to compute weighted combinations of the value representations [37]. Consequently, the representation generated for each input element is conditioned on information from the entire sequence rather than only on adjacent elements.

Rather than using a single attention operation, the Transformer employs multi-head attention. Multiple attention heads operate in parallel using separate query, key, and value projection matrices. Each head therefore processes the input within a different learned representation subspace. The outputs of the individual heads are concatenated and passed through a final linear transformation before moving to the next network layer. This structure allows the model to represent different relationships within the input sequence simultaneously.

Attention operations do not inherently preserve sequence order. Therefore, the model adds positional information to the input and output embeddings before they enter the encoder and decoder. The original Transformer uses sinusoidal positional encoding, in which each position is represented by a predefined combination of sine and cosine functions. Positional encoding lets the model distinguish elements at different positions while maintaining parallel processing. Modified Transformer architectures may also use learnable positional representations.

The computational and memory requirements of standard self-attention depend strongly on sequence length. Because it calculates an attention score for every pair of sequence elements, its complexity increases quadratically with sequence length. This can become computationally demanding when processing long sequences and may require reducing the number of input elements before passing them to the Transformer.

For vibrational spectroscopy, the input is an ordered sequence of numerical intensities sampled along a wavenumber or wavelength axis. This sequence can be represented using individual spectral variables or divided into short segments of neighboring variables, commonly termed spectral patches [45]. Each variable or patch is mapped by an embedding layer to a vector of fixed dimensionality, forming the sequence of tokens processed by the Transformer. Non-overlapping patches divide the spectrum into separate segments, whereas sliding-window tokenization can produce overlapping patches by moving the window along the spectral axis with a step smaller than its length. Positional information is added to the embeddings to represent the location of each token within the spectrum. Depending on the analytical objective, the encoder output can be connected to a classification or regression head, while tasks involving sequence generation may also use a decoder.

In full self-attention, attention scores are calculated for every pair of tokens, so the computational cost of these pairwise operations increases quadratically with the number of tokens [37]. Even when spectral variables are highly correlated, full self-attention still calculates attention scores for every pair of tokens. Grouping spectral variables into patches can shorten the token sequence, while local-window and sparse attention restrict interactions to neighboring tokens or selected connections [46,47]. Specific implementations of these approaches are discussed in the following application sections.

## 3. Applications of Transformer Models in Vibrational Spectroscopy

**Figure 3:**
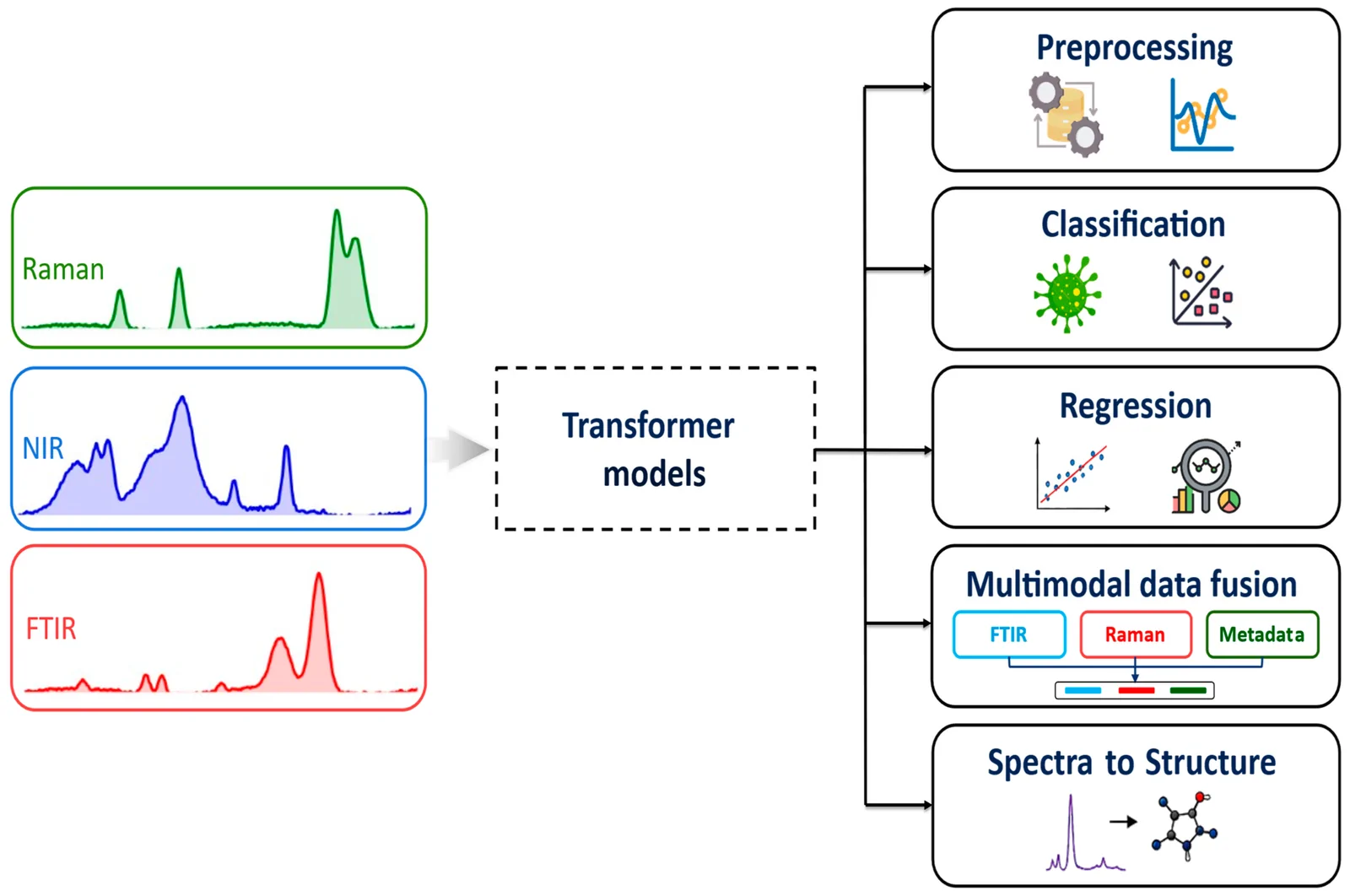
Overview of Transformer applications in vibrational spectroscopy.

The studies reviewed in this section can be grouped into five main application areas: spectral preprocessing, classification, regression and quantitative analysis, multimodal data fusion, and spectra-to-structure prediction. Figure 3 presents a conceptual overview of these application areas.

Vibrational spectra, including Raman, mid-infrared, and near-infrared spectra, are first converted into representations suitable for Transformer processing. Depending on the analytical task, the resulting spectral tokens may be processed with an encoder-only Transformer, an encoder–decoder architecture, or a hybrid model that combines Transformer blocks with other neural-network components. The learned representations are subsequently used for spectral correction or reconstruction, categorical prediction, estimation of continuous properties or concentrations, integration of complementary data modalities, or prediction of molecular information from spectra. The following sections review these application categories and compare the corresponding model architectures, datasets, validation strategies, and reported performance.

### 3.1. Preprocessing

Raw vibrational spectra are frequently affected by baseline drift, random noise, overlapping bands, and instrumental broadening, which can obscure chemically relevant features and reduce the reliability of subsequent analyses. Parametric methods such as modified polynomial fitting (ModPoly), improved modified polynomial fitting (IModPoly), and adaptive iteratively reweighted penalized least squares (airPLS) are commonly used for Raman baseline correction. However, their performance can depend strongly on parameter selection and the characteristics of the measured spectrum [48]. The preprocessing studies reviewed in this section are summarized in Table S1.

**Figure 4:**
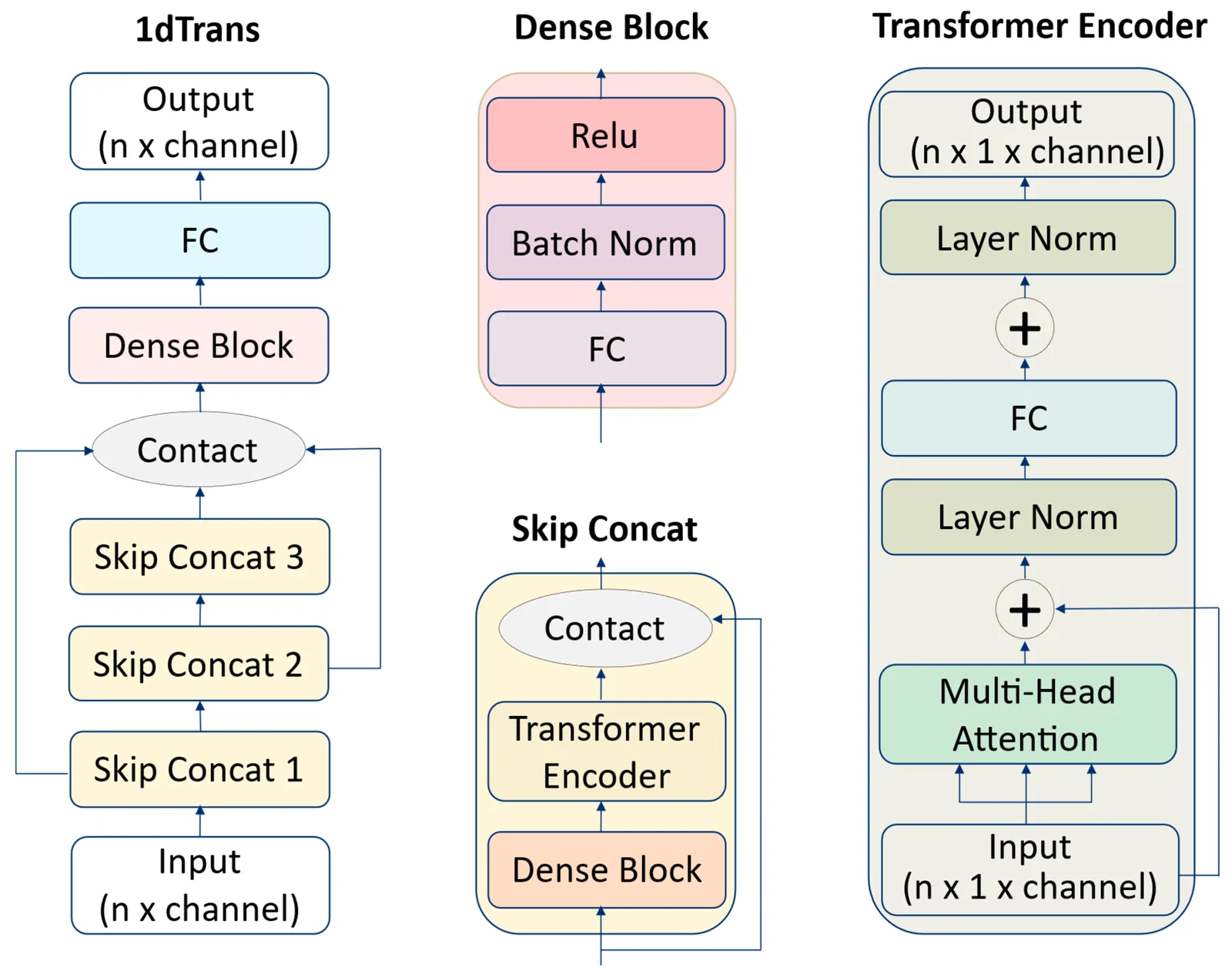
Architecture of the one-dimensional Transformer (1dTrans) for Raman spectral baseline estimation. Adapted from Ref. [48].

Transformer-based preprocessing methods provide a data-driven alternative by learning relationships between the degraded and reference spectra. Zhao et al. developed a one-dimensional Transformer, termed 1dTrans, for baseline estimation in Raman spectra [48]. The model combines dense blocks, skip concatenation, and Transformer encoders to integrate multilevel features while capturing dependencies across the spectral sequence (Figure 4). Ground-truth baselines were manually constructed for Raman spectra of eight biological materials by combining carefully optimized outputs from ModPoly, IModPoly, and airPLS. In the reported evaluation, 1dTrans achieved an MAE of 1.22 × 10^−5^ and a spectral angle mapper value of 0.0069, outperforming CNN, ResUNet, and the three parametric baseline-correction methods. These results demonstrate the potential of Transformer-based models to reduce the dependence of Raman baseline correction on manually selected parameters.

Infrared spectral preprocessing primarily focuses on deconvolution, which aims to recover spectral peaks distorted by instrumental broadening, band overlap, and noise. Gao et al. introduced a masked pre-training Transformer that uses patch embedding and random spectral masking to learn contextual representations before reconstructing degraded IR spectra [49]. Subsequent work introduced the sparse self-attention Transformer (SST), which combined sparse attention, convolutional operations, and feature distillation to model long spectral sequences with reduced computational demand [46]. Its generative decoder reconstructs the complete spectrum in a single forward pass and was reported to preserve peak structures under Gaussian, Lorentzian, and mixed broadening conditions.

Gao et al. proposed the auto-correlation multi-head attention Transformer (AMTrans) for IR spectral deconvolution [50]. Instead of conventional scaled dot-product attention, AMTrans uses an auto-correlation mechanism implemented through fast Fourier transforms and delay aggregation to identify and combine recurrent dependencies within spectral sequences. The model was trained on 4000 synthetic IR spectra containing Gaussian, Lorentzian, and mixed broadening with additive noise and was compared with semi-blind deconvolution, unpaired learning, GAN, self-paced learning, and DSPNet approaches. AMTrans produced lower RMSE and higher correlation-based metrics across the investigated degradation conditions. Evaluation using spectra acquired from conventional and high-performance instruments also indicated its ability to preserve peak position, shape, and intensity under real instrumental degradation.

Collectively, these studies show that Transformer-based preprocessing is evolving from Raman baseline estimation toward infrared spectral deconvolution and restoration. Nevertheless, most available models have been trained predominantly on simulated or manually constructed reference data. Their robustness to previously unseen samples, instruments, noise distributions, and acquisition conditions remains insufficiently established. Future studies should therefore prioritize cross-instrument validation, physically realistic degradation models, and quantitative assessment of peak preservation to ensure that preprocessing does not introduce artificial spectral features.

### 3.2. Classification

Classification is one of the most extensively investigated applications of Transformer models in vibrational spectroscopy, with examples spanning pharmaceutical analysis, microbial identification, biomedical diagnosis, food authentication, environmental monitoring, and materials characterization. Conventional chemometric and machine-learning methods, including PLS-DA, LDA, and SVM, remain effective for classifying many spectral datasets. However, their performance may depend strongly on preprocessing, feature selection, and the similarity between calibration and test samples. Transformer-based models provide an alternative approach in which discriminative representations can be learned directly from complete spectra or spectral segments.

Recent Transformer-based classification studies can be broadly divided into two architectural groups: (i) encoder-based Transformer architectures and (ii) hybrid Transformer architectures. Encoder-based models primarily learn global representations directly from spectral sequences, whereas hybrid models combine the local feature-extraction capability of CNNs with the global modeling capability of Transformers.

#### 3.2.1. Encoder-Based Transformer Architectures

**Figure 5:**
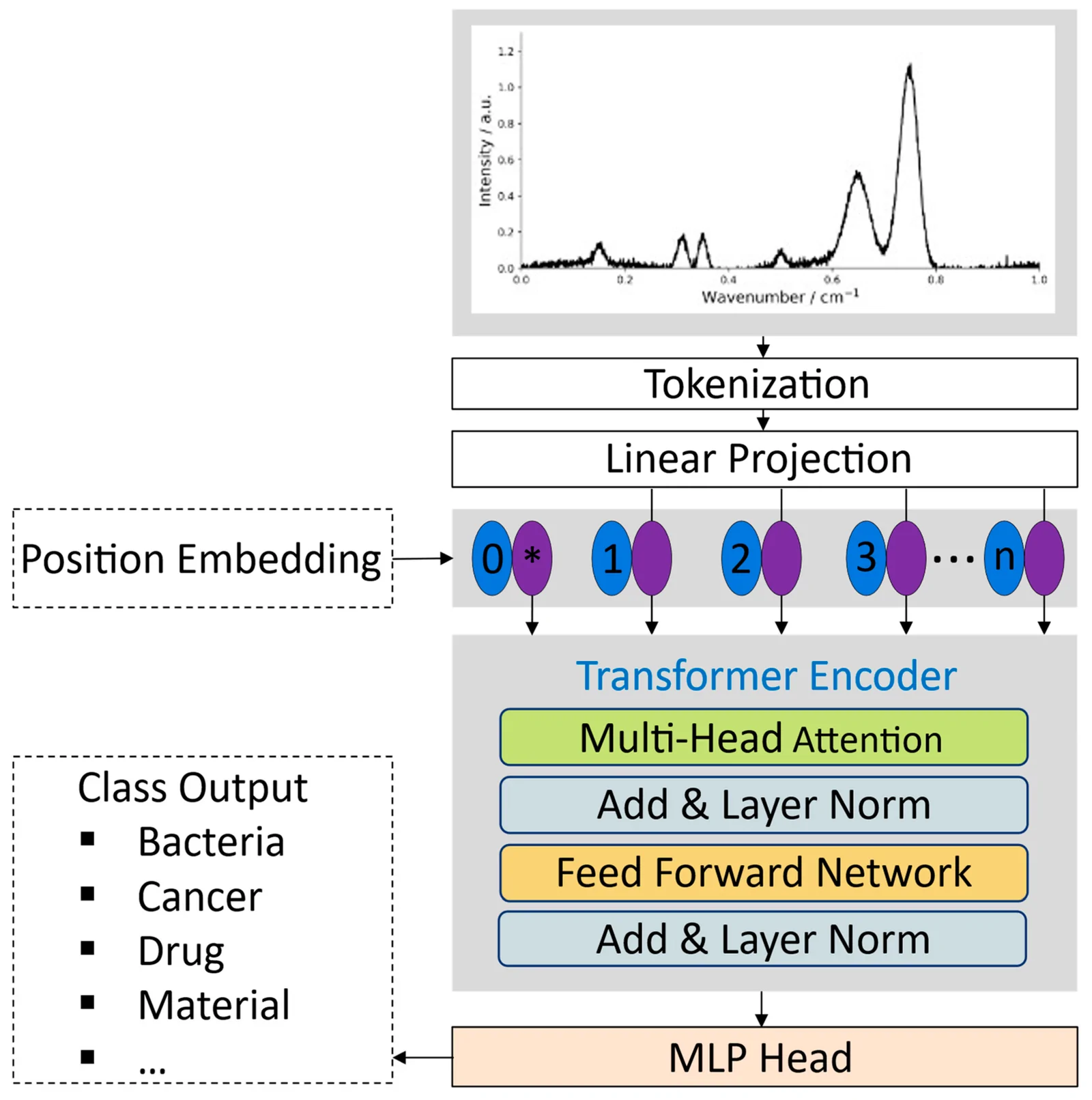
General workflow of an encoder-based Transformer for vibrational spectral classification. The input spectrum is converted into spectral tokens with positional information and processed by stacked Transformer encoders. A class token or pooled representation is subsequently passed to an MLP or fully connected head to generate the classification output. The asterisk (*) denotes the class token.

A commonly used architecture for vibrational spectral classification combines a Transformer encoder with a multilayer perceptron (MLP) or fully connected classification head (Figure 5). The input spectrum is represented as a sequence of spectral variables or patches and projected into an embedding space. Positional information preserves the spectral-axis ordering, and stacked Transformer encoder blocks process the resulting tokens. Finally, the classification head receives a class token or pooled encoder representation. Although the embedding and tokenization procedures vary among studies, this general workflow has been used for NIR, Raman, SERS, and FTIR classification [45,51,52,53,54,55].

SpectraTr represents an example of this architecture in vibrational spectroscopy [45]. The model divided NIR spectra into patches and processed them using a Transformer encoder and MLP layers. It achieved classification accuracies of 100% and 99.52% for datasets representing seven and eighteen pharmaceutical manufacturers, respectively, using a random 80:20 training–test split. An accuracy of 96.97% was also reported on a public dataset without dedicated spectral preprocessing. A similar encoder–MLP architecture was applied to single-cell Raman spectra from eight deep-sea bacterial strains and achieved an average accuracy of 97.3% using fivefold cross-validation [51]. The folds were constructed from spectra within each strain, without specifying separation by independent bacterial cultures. These studies demonstrated the feasibility of combining spectral tokenization, Transformer encoding, and MLP-based prediction for one-dimensional vibrational spectra.

The same general architecture was subsequently extended to bacterial identification using SERS, melanoma detection with the Raman Transformer, drug identification in biological samples using TMNet, esophageal-cancer grading, and FTIR materials classification through transfer learning [52,53,54,55,56]. In the FTIR transfer-learning study, one original spectrum per molecule was reserved for testing before augmentation, while the remaining spectra were augmented and subsequently divided into training and validation subsets [55]. More specialized models introduced multiscale fragmentation, categorical spectral embeddings, shifted-window attention, and chemistry-informed spectral processing [47,57,58,59,60]. These modifications were designed to improve the representation of local spectral patterns, robustness to noise, generalization across samples, or interpretation of the spectral regions contributing to classification. Encoder-based Transformers were also applied to raw SERS maps, infrared hyperspectral images, and salivary FTIR spectra, extending their use from individual one-dimensional spectra to spatial–spectral classification [61,62,63].

Several studies addressed limited training data and unfamiliar classes through complementary strategies. LSGAN- and WGAN-assisted pipelines generated synthetic Raman or SERS spectra before Transformer classification [64,65,66], whereas an open-set model combined classification and reconstruction to reject bacterial species absent from the training set [67]. These models are considered augmentation- or reconstruction-assisted encoder architectures.

Despite the high accuracies reported in many individual applications, controlled comparisons have produced more variable results. A replicate-aware bacterial Raman study reported that a Transformer outperformed conventional chemometric pipelines [68]. In contrast, a glioblastoma study found that a 1D-CNN significantly outperformed a 1D Vision Transformer while requiring approximately one-tenth of its computational time [69]. A recent multi-dataset benchmark similarly showed that Transformer models were not consistently superior to Raman-specific CNN architectures [70]. In that benchmark, hyperparameter selection and early stopping used validation subsets, with final performance evaluated on separate test sets. These findings indicate that the effectiveness of encoder-based Transformers depends strongly on dataset size, preprocessing, validation design, and the degree of distribution shift.

#### 3.2.2. Hybrid Transformer Architectures

**Figure 6:**
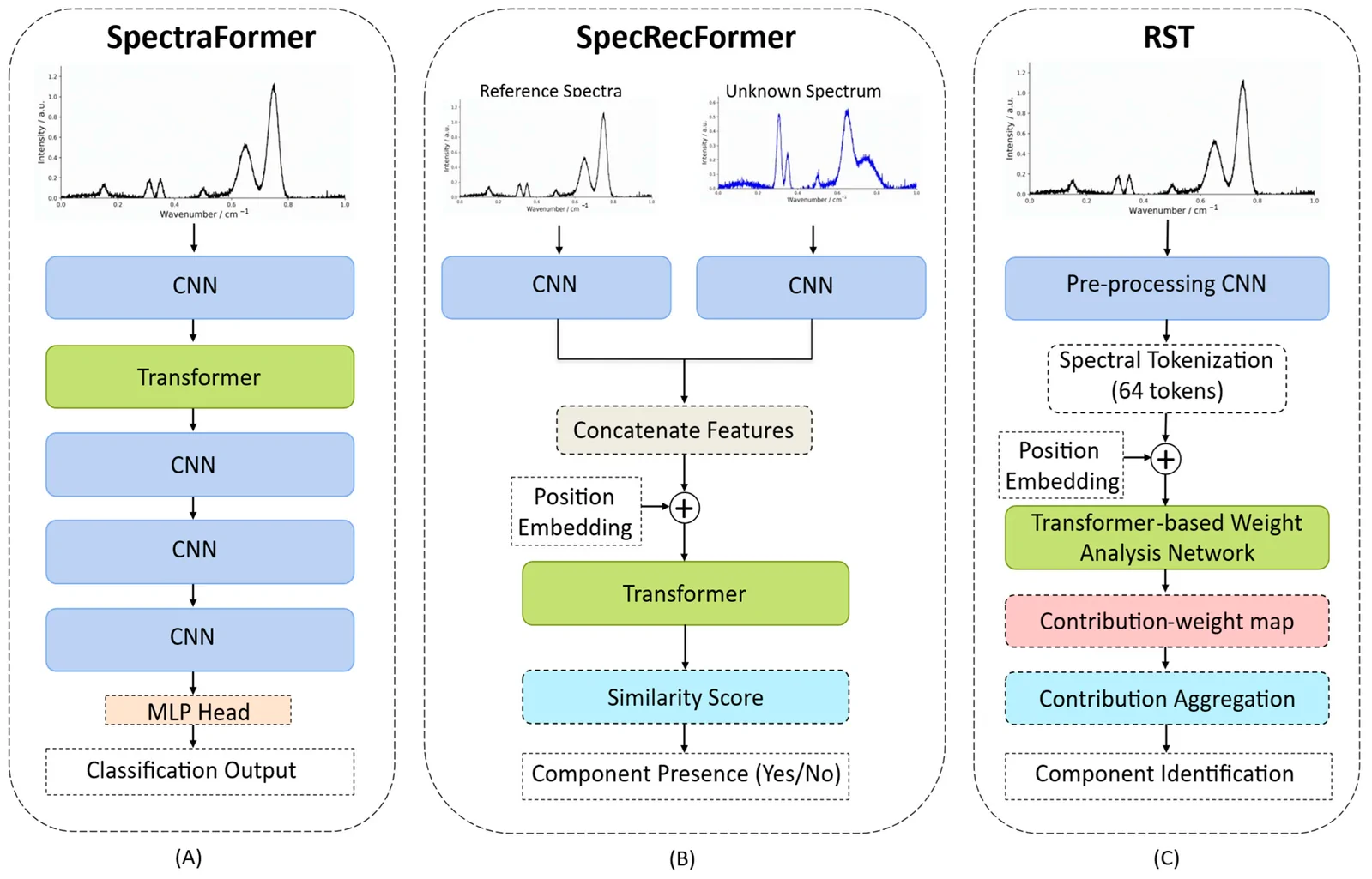
Hybrid architectures integrating Transformer models for vibrational spectral analysis. (**A**) Schematic representation of the Spectraformer architecture based on Ref. [71]. (**B**) Schematic representation of SpecRecFormer, in which features extracted from a single-component reference spectrum and an unknown mixture spectrum are concatenated and processed by a Transformer to predict the presence of the reference component, based on Ref. [72]. (**C**) Raman Spectral Translation (RST) network for component identification in Raman mixtures. Spectral features extracted by a preprocessing convolutional neural network (CNN) are processed by a Transformer-based weight-analysis network to generate a contribution-weight map. The contributions across the spectral segments are then aggregated for component identification, based on Ref. [73].

Hybrid CNN–Transformer models combine convolutional feature extraction with attention-based representation learning. The CNN components identify local spectral characteristics, such as peak shapes, intensities, and neighboring band patterns, whereas the Transformer components model relationships among features originating from different spectral regions. However, the reported models arrange these components differently. Figure 6 presents three representative integration strategies used for spectral classification and mixture-component identification.

In Spectraformer, shown in Figure 6A, a CNN layer first processes the input spectrum before it enters a Transformer module [71]. The Transformer output is subsequently refined through several additional convolutional layers, and the resulting representation is passed to an MLP head for classification. Thus, the Transformer is embedded within a predominantly convolutional pipeline rather than being placed only after the complete CNN feature extractor. This architecture was evaluated using compact NIR spectra to classify barley, chickpea, and sorghum varieties, with maximum reported accuracies of approximately 85%, 95%, and 86%, respectively.

SpecRecFormer employs the dual-branch architecture shown in Figure 6B [72]. A single-component reference spectrum and an unknown mixture spectrum are independently processed by two CNN branches. The extracted representations are concatenated and passed to a Transformer, which models relationships between reference and unknown spectral features. The final similarity score indicates whether the reference compound is present in the mixture. SpecRecFormer achieved accuracy of 93.75%, 89.21%, and 93.63% on standard, expanded, and SERS polycyclic aromatic hydrocarbon mixture datasets, respectively. This model therefore performs reference-guided component identification rather than conventional single-label classification.

The Raman Spectral Translation (RST), presented in Figure 6C, uses a preprocessing CNN to convert the input Raman spectrum into a set of spectral feature vectors [73]. A Transformer-based weight-analysis network then analyzes these features and estimates the contribution of individual spectral regions. The resulting contribution map is subsequently aggregated to generate the final component predictions. In addition to identifying multiple components within a mixture, this design provides information about the spectral regions contributing to each prediction.

Other hybrid architectures follow the same general principle but employ different feature-extraction and prediction modules. A CNN–Transformer framework classified seventeen environmentally sourced microplastic types from FTIR spectra with 95.77% accuracy on a validation subset obtained from an 80:20 training–validation split [74]. External validation on independently collected data was not reported. A two-stage CNN–Transformer pipeline achieved 85.0% accuracy and a weighted F1 score of 85.0% in classifying thirty bacterial species from Raman spectra [75]. A hybrid 1D-CNN–Transformer classified four skin-lesion categories, including malignant melanoma, basal cell carcinoma, squamous cell carcinoma, and benign lesions, achieving a mean accuracy of 96.8% and a macro-averaged AUC of 0.987 under tenfold cross-validation [76]. Hyperparameter tuning used a validation subset within the training folds, but patient-level grouping was not specified.

DeepMIR combined convolutional feature extraction with a Transformer encoder and achieved a mean test accuracy of 99.2% across ten runs, with the best-performing run reaching 99.5%, for targeted component identification from mid-infrared spectra of chemical mixtures [77]. The reference library was divided into training, validation, and test subsets at an 80:10:10 ratio before synthetic mixtures were generated independently within each subset. Further evaluation used experimentally measured liquid, solid, and textile mixtures. SMART-NIR further extended this strategy by integrating multikernel feature extraction, a modified Vision Transformer, and a Kolmogorov–Arnold Network classifier, achieving a mean classification accuracy of 99.24% under stratified fivefold cross-validation on 1028 spectral measurements [78]. Grouping by physical specimen was not specified in the reported evaluation protocol.

Although these models are collectively categorized as hybrid architectures, their analytical objectives differ. Spectraformer and the CNN–Transformer models developed for microplastics, bacteria, and skin cancer perform conventional classification. SpecRecFormer, RST, and DeepMIR instead address reference-guided or multilabel component identification in spectral mixtures. Their reported results should therefore be interpreted according to the corresponding analytical task and validation design rather than compared directly using classification accuracy alone.

Collectively, these studies demonstrate the broad applicability of Transformer-based models to vibrational spectral classification, encompassing conventional single-label classification, hierarchical and open-set recognition, and multilabel identification of mixture components. As summarized in Table 1, these approaches have been applied to Raman, SERS, FTIR, and NIR data across biological, medical, pharmaceutical, environmental, forensic, agricultural, and materials-science applications. Their architectures range from conventional spectral encoders and Vision Transformers to hybrid CNN–Transformer models and networks incorporating data augmentation, reconstruction, or domain adaptation.

Future classification studies should prioritize sample-independence and replicate-aware data partitioning to minimize information leakage and provide a more realistic assessment of model generalization. When biological replicates represent independent experimental units, leave-one-replicate-out validation can be used to withhold all spectra from one replicate for evaluation in each fold [79]. Original spectra should be partitioned before augmentation, with each original spectrum and its augmented variants retained within the same subset [80]. These validation principles apply to both Transformers and the baseline models used for comparison. External validation across instruments, laboratories, and acquisition conditions is also needed to establish the transferability of Transformer-based spectral models. Preprocessing, augmentation, and validation procedures should be reported transparently, and newly proposed architectures should be compared with appropriately optimized chemometric and CNN-based baselines. Model interpretability and computational efficiency should also be considered when evaluating these models for practical spectroscopic applications. Differences in validation design and the independence of test samples therefore limit direct comparisons of reported classification performance across studies.

### 3.3. Regression and Quantitative Analysis

Regression and quantitative analysis represent important applications of vibrational spectroscopy, enabling continuous chemical and physical properties to be estimated directly from Raman, IR, and NIR measurements. Conventional calibration methods, particularly partial least-squares regression and support vector regression, remain established benchmarks for these tasks [33]. More recently, Transformer-based regression models have expanded quantitative spectral analysis by learning informative representations across the spectral range. Their applications include Vis–NIR prediction of soil properties [81], quantification of components in Raman mixtures [82], NIR-based assessment of protein content [83], and online monitoring of fermentation processes [84]. These studies illustrate the growing role of Transformer architectures in quantitative vibrational spectroscopy. Table S2 summarizes the studies reviewed in this section on regression and quantitative analysis.

#### 3.3.1. Transformer-Based Regression Models

Transformer-based regression models have been developed to estimate continuous chemical and physical properties from vibrational spectra. Most of these models employ a Transformer encoder to learn relationships among ordered spectral variables or spectral patches, followed by a regression head that generates one or more quantitative outputs. Recent studies have adapted this framework through application-specific spectral representations, incorporation of physicochemical knowledge, and integration with portable spectrometers.

ProTformer is a Transformer-based model designed specifically for quantitative NIR analysis [83]. The model was developed to predict the protein content of 112 lablab bean genotypes and employed multi-head self-attention to extract complementary information from different NIR regions. ProTformer achieved an RSQ of 0.977 and an RPD of 13.92, outperforming both 1D-CNN and modified partial least-squares regression models. This study showed that a Transformer-centered model could improve prediction of an important nutritional trait relative to the selected convolutional and chemometric benchmarks.

Analytical knowledge was incorporated more explicitly in the Physicochemical-Informed Spectral Transformer (PIST) [85]. This model combined a physicochemical-informed block for domain adaptation with a feature-embedding block for global spectral representation. When applied to chemical oxygen demand prediction in surface-water samples, PIST achieved an R^2^ of 0.9008 and reduced RMSE by 45.20% and 29.38% relative to SVR and CNN models, respectively. The spectra used in this study span both the UV–Vis and short-wave near-infrared (SWNIR) regions. UV–Vis probes electronic transitions, whereas only the SWNIR component falls within the vibrational scope of this review.

A broader comparison of NIR regression methods was performed for protein prediction in maize powder [86]. The Transformer generally outperformed ResNet-18 across the evaluated preprocessing strategies, with its best result obtained using first-derivative and SNV preprocessing. However, a PLSR model using first-derivative and MSC preprocessing followed by SPA wavelength selection achieved the best overall performance (R_p = 0.927), (RMSE_p = 0.301), and (RPD = 2.502). This result indicates that established chemometric models can remain competitive for small, strongly collinear spectral datasets.

Other Transformer-centered studies converted Vis–NIR or MIR soil spectra into two-dimensional GADF representations before applying Swin Transformer regression [81,87]. Transformer regression was also coupled with miniature NIR spectroscopy to nondestructively determine quality-related compounds in *Angelica sinensis* [88]. More recently, SpecTran introduced multiscale patch embedding, spectral attention, and multipath feature fusion to simultaneously predict moisture, starch, oil, and protein in corn [89].

Overall, these studies demonstrate that Transformer-based regression can support quantitative analysis across agricultural, environmental, and food-related spectroscopy. Its performance, however, remains dependent on dataset size, spectral representation, preprocessing, and the complexity of the underlying spectral–property relationship.

#### 3.3.2. Hybrid Transformer-Based Regression Models

Hybrid Transformer-based regression models combine attention-based global modeling with complementary components that address specific limitations of spectral datasets. Depending on the analytical problem, these components may include convolutional layers to extract local spectral patterns, recurrent networks for sequential feature learning, or generative models to expand limited calibration datasets.

RamanFormer is a hybrid Transformer-based model developed for quantitative Raman analysis [82]. The input Raman spectrum is divided into non-overlapping patches, projected into an embedding space, and processed by three Transformer encoder layers. The encoded features then pass through one-dimensional convolutional layers, global average pooling, and linear regression layers to estimate the relative amounts of individual mixture components. The Transformer encoders capture relationships among overlapping spectral regions, whereas the convolutional layers refine local features in the encoded representation. RamanFormer was evaluated using binary and ternary mixtures of methanol, isopropyl alcohol, and ethanolamine and achieved a mean absolute error of 1.4% and a root mean squared error of 1.6%. It outperformed least-squares fitting, MLP, VGG11, and ResNet50 models and maintained reliable predictions under noisy measurement conditions.

A different hybridization strategy was adopted for online NIR monitoring of hyaluronic acid fermentation [84]. In this framework, a Wasserstein generative adversarial network with gradient penalty (WGAN-GP) generated synthetic spectra to expand the limited calibration dataset, while an improved Transformer predicted hyaluronic acid concentration and molecular weight. The combined framework achieved better predictive accuracy and generalization than PLSR, 1D-CNN, and LSTM models on independent test sets. Its main contribution was integrating generative spectral augmentation with attention-based regression, providing a practical approach for quantitative process monitoring when representative calibration samples are difficult to obtain.

SpecTransformer combined recurrent and attention-based feature learning for the non-destructive determination of thiophanate-methyl in cherry tomatoes using a handheld spectrometer [90]. Wavelet-based input enrichment and spectral preprocessing were followed by a GRU-based residual block and a Transformer encoder. The recurrent component extracted sequential spectral features, while multi-head self-attention modeled relationships across the measured spectral bands. SpecTransformer achieved an R^2^ of 0.91 and an RMSE of 1.05, with a reported detection limit of 0.2 g/L. The study is particularly notable for integrating a hybrid regression model with a compact spectrometer, cloud computing, and a mobile application for on-site analysis.

Collectively, these studies represent three distinct forms of hybridization: Transformer–CNN feature integration, GAN-assisted calibration expansion, and GRU–Transformer sequential modeling. Such combinations can improve local feature extraction, robustness under limited-data conditions, and compatibility with portable instrumentation. However, the increased architectural complexity makes it more difficult to determine the individual contribution of each component.

### 3.4. Multimodal Data Fusion

Different spectroscopic techniques provide complementary information about the same sample. The fusion approaches reviewed in this section combine Raman, IR, or NIR spectra with complementary analytical measurements or sample metadata. Raman and IR spectroscopy probe different vibrational selection rules, whereas elastic scattering, LIBS, NMR, metabolomics, and sample metadata can provide additional morphological, elemental, structural, or contextual information. Transformer-based fusion models can integrate these heterogeneous sources while learning both modality-specific features and interactions between modalities. Current applications include disease diagnosis, tissue and polymer classification, molecular identification, and quantitative analysis. The multimodal studies reviewed in this section are summarized in Table S3.

**Figure 7:**
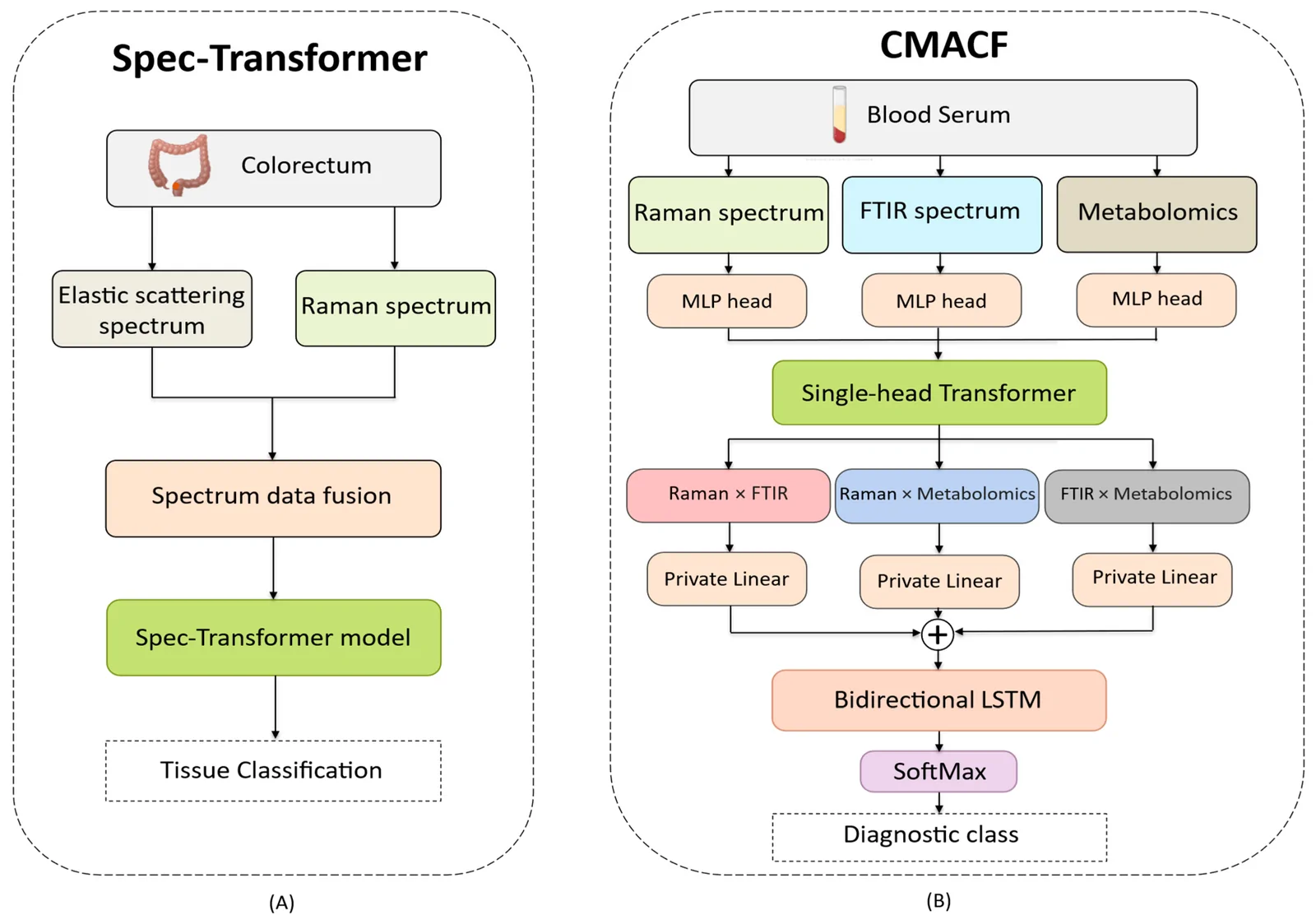
Transformer-based multimodal data-fusion strategies. (**A**) Fusion of Raman and elastic-scattering spectra using the Spec-Transformer model for binary classification of normal and cancerous colorectal tissues [92]. (**B**) Conceptual overview of the CMACF workflow for diagnostic classification using serum-derived Raman, FTIR, and metabolomics data [91].

CMACF was developed to diagnose systemic lupus erythematosus using Raman and FTIR spectra together with metabolomics data from serum samples [91]. Separate multilayer perceptron (MLP) heads first encoded the three modalities, after which a single-head Transformer modeled their cross-modal relationships. Pairwise Raman–FTIR, Raman–metabolomics, and FTIR–metabolomics interactions were subsequently formed through cross-multiplication, processed by pair-specific linear mappings, subjected to bimodal-interaction similarity validation, and concatenated. The fused representation was then processed by a bidirectional long short-term memory (BiLSTM) network for diagnostic classification. CMACF achieved an accuracy of 99.44% and an area under the receiver operating characteristic curve (AUC) of 99.98%. Figure 7B summarizes the principal stages of the architecture.

The Spec-Transformer model used a different fusion strategy by combining Raman and elastic scattering spectra for colorectal-tissue classification [92]. Raman spectra provided biochemical information, whereas elastic scattering contributed complementary information about tissue morphology. Following multimodal spectral processing and fusion, the combined representation was analyzed using the composite Spec-Transformer model, which achieved a classification accuracy of at least 97%. Although elastic scattering is not itself a vibrational technique, this study demonstrates how Raman spectroscopy can be complemented by another optical measurement. Figure 7A shows the simplified workflow.

Transformer-based fusion has also been investigated using multiple analytical spectra. Xu et al. combined FTIR, Raman, and LIBS measurements to identify recyclable polymers [93]. The model used Transformer-based analysis for multimodal fusion and cross-modal spectral generation, while a genetic algorithm optimized the model hyperparameters. Multimodal fusion achieved an accuracy of 97.69%, exceeding the best single-modality model by 9.74 percentage points, and the optimized model achieved an accuracy above 99.23% on the independent test set. Wu et al. extended the TranSpec framework to integrate IR, Raman, and NMR spectra for molecular identification [94]. These complementary spectral inputs improved molecular recognition relative to individual modalities, illustrating the potential of multimodal learning to distinguish structurally similar compounds.

More recent studies have introduced increasingly elaborate fusion architectures. The Trinity-Branch Parallel Fusion and Supervised Enhancement Network (TFS-Net) employed separate encoders for Raman, IR, and metabolomics data, followed by pairwise cross-modal attention, dual-tower similarity supervision, and a Transformer encoder for global feature modeling [95]. The model achieved 95.82% accuracy for celiac disease diagnosis under five-fold cross-validation. In quantitative analysis, Wang et al. developed an interpretable CNN–Transformer multimodal hierarchical fusion network that combined UV–Vis–NIR spectral features with auxiliary factors such as sample origin, cultivar, and temperature [96]. A one-dimensional CNN extracted local spectral features, whereas the Transformer modeled interactions between the spectral representation and encoded auxiliary variables. For mango dry-matter prediction, multimodal fusion reduced the validation and test RMSE values from 0.984 and 1.03 to 0.577 and 0.613, respectively.

Overall, these studies show that Transformer-based fusion is progressing toward cross-modal attention, pairwise interaction modeling, and hierarchical integration of spectra with complementary analytical or contextual information. Further progress will depend on well-aligned multimodal datasets and models that remain reliable when individual modalities are noisy or unavailable.

### 3.5. Spectra-to-Structure Prediction

Spectra-to-structure prediction aims to infer atomic connectivity from a vibrational spectrum and represent the resulting molecule, commonly as a Simplified Molecular Input Line Entry System (SMILES) sequence. Recent Transformer-based studies have followed two principal strategies: direct SMILES generation and retrieval of candidate structures based on spectrum–molecule similarity. The studies considered here use IR as the only measured spectral modality, with either simulated or experimental spectra, although some additionally incorporate molecular formulas or structural representations; they are therefore discussed separately from multimodal spectral-fusion approaches. Table S4 summarizes the studies reviewed in this section on molecular generation and candidate retrieval.

Alberts et al. introduced an encoder–decoder Transformer for molecular structure elucidation from IR spectra and molecular formulas [97]. The model was pretrained on 634,585 simulated spectra and fine-tuned on 3453 experimental spectra, achieving Top-1 and Top-10 accuracies of 44.4% and 69.8%, respectively. This study demonstrated the feasibility of directly generating complete molecular structures as SMILES sequences, although the molecular formula provided additional constraints on the prediction space.

Kanakala et al. proposed the Spectra and Molecule Encoder Network (SMEN), which combined a Transformer spectrum encoder with a molecular graph encoder and used contrastive learning to rank candidate structures [98]. On simulated IR spectra derived from the QM9 dataset, SMEN achieved approximately 81% Top-1 and 99% Top-10 accuracy when ranking candidate structures from a predefined molecular library. These values describe a retrieval task rather than direct molecular generation. In the same study, the learned spectral representation was also coupled to a generative Transformer decoder for direct SMILES prediction.

**Figure 8:**
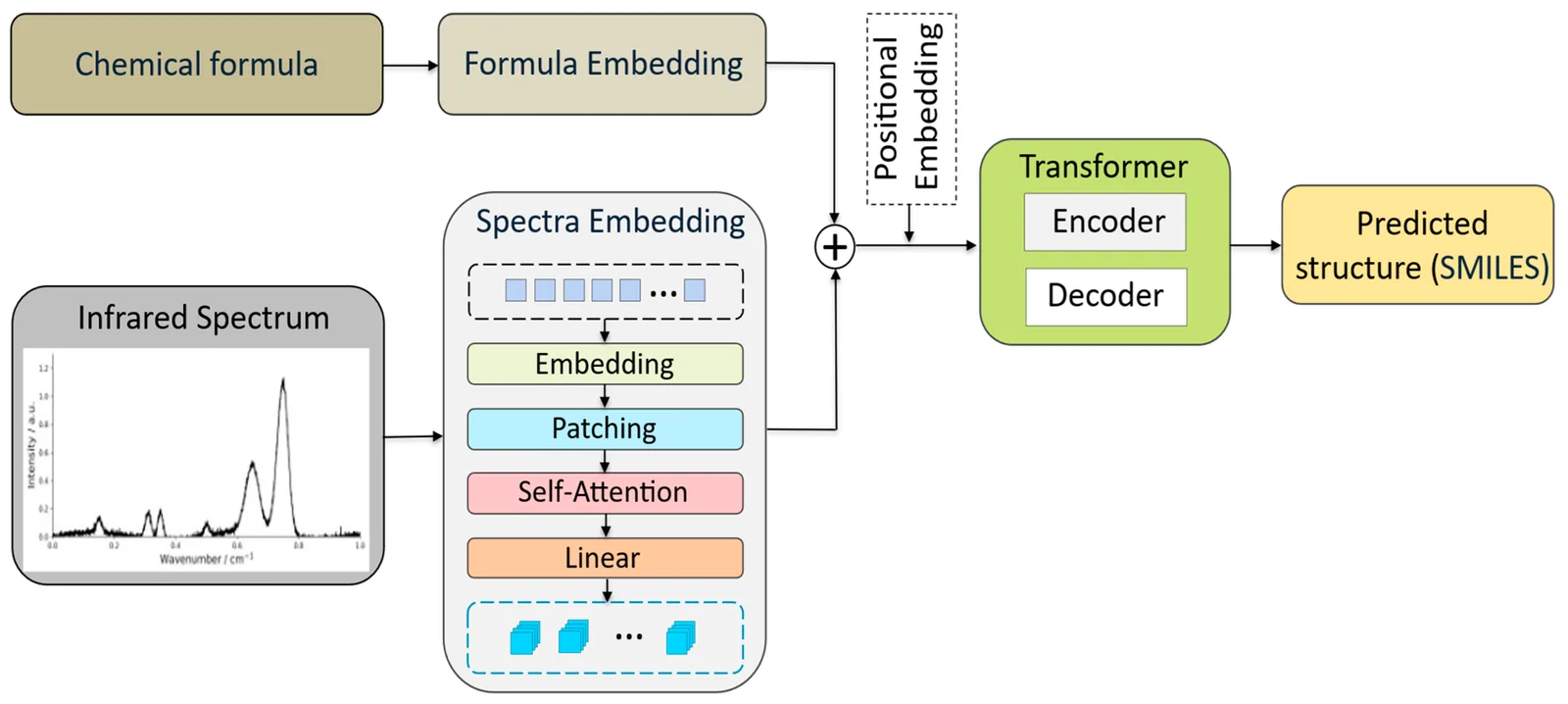
Conceptual workflow of the patch-based encoder–decoder Transformer proposed by Wu et al. for direct molecular structure generation from an infrared spectrum and molecular formula. Spectral patches and molecular formula embeddings, together with positional information, are processed by the Transformer architecture to generate the predicted molecular structure as a Simplified Molecular Input Line Entry System (SMILES) sequence [39].

Wu et al. introduced a patch-based self-attention embedding layer to retain detailed spectral information, together with a peak-aware augmentation method termed Adaptive Noise [39]. The model used IR spectra and molecular formulas as inputs and achieved 55% Top-1 accuracy on the experimental NIST dataset. Figure 8 presents the patch-based generative architecture proposed by Wu et al.

Alberts et al. subsequently refined their original Transformer by modifying the model architecture and spectral representation, adding augmentation, and using formula-constrained decoding strategies [99]. The improved model achieved Top-1 and Top-10 accuracies of 63.79% and 83.95%, respectively. Zhang and Ha later adapted the Contrastive Captioner (CoCa) architecture by combining a contrastive objective for spectrum–molecule alignment with a captioning objective for autoregressive SMILES generation [100]. Unlike retrieval-based approaches, this model directly generates molecular structures from IR spectra without relying on a predefined candidate library.

Overall, these studies demonstrate the potential of Transformers for both molecular generation and candidate retrieval from IR spectra. However, differences in prediction tasks, input information, datasets, and evaluation protocols prevent direct comparisons of reported performance. Current models also frequently rely on simulated data, relatively small molecules, known molecular formulas, or candidate libraries. Future studies should therefore prioritize diverse experimental datasets, external validation, and chemically valid predictions with reliable uncertainty estimates.

## 4. Challenges and Future Perspectives

Transformer models have shown potential in preprocessing, classification, quantitative analysis, multimodal fusion, and spectra-to-structure prediction. However, many studies have used relatively small, application-specific, augmented, or simulated datasets, which may not fully represent experimental variability. A large number of trainable parameters can increase the risk of overfitting when the number and diversity of independent training samples are limited. Numerous spectral variables or repeated measurements do not necessarily compensate for this limitation, particularly when they contain strongly correlated or redundant information. Model complexity should therefore be considered in relation to the available data. The competitive or superior performance of CNNs and conventional chemometric methods in several reviewed studies further supports the need for comparisons with appropriately optimized baseline models. The limited availability of standardized public datasets also makes direct comparison between different architectures difficult.

Model generalization remains another important challenge because spectral distributions can vary across instruments, laboratories, samples, and acquisition conditions. Random spectrum-level splitting may also produce optimistic results when spectra from the same sample or replicate appear in both training and test sets. Future studies should therefore prioritize sample-independent validation, external datasets, and transparent reporting of preprocessing and data-partitioning procedures. Multimodal models should additionally be evaluated when one modality is missing or degraded, since most existing fusion studies assume that all required inputs are available.

Attention maps can identify spectral regions associated with model predictions, but attention weights alone do not necessarily fully explain model behavior. Complementary methods and established vibrational-band assignments should support their interpretation. Uncertainty estimation is also needed, particularly in biomedical diagnosis, quantitative prediction, and molecular-structure elucidation, where most current studies primarily report point-performance metrics. Spectra-to-structure models still largely depend on simulated data, known molecular formulas, relatively small molecules, or predefined candidate libraries.

Computational efficiency should also be considered because the cost of full self-attention increases quadratically with the number of spectral tokens. Sparse attention, spectral patching, and hierarchical processing can help reduce this burden, although practical computational requirements also depend on model size, training strategy, and hardware. Alberts et al. reported approximately 35 h of training on a V100 GPU for their spectra-to-structure model [97]. RamanFormerSSL, with 8.97 million parameters, achieved inference throughputs of 69,119 spectra/s on an A100 GPU and 7237 spectra/s on a 16-core CPU [101]. Such computational requirements should be considered alongside the analytical benefit. DeepMIR processed 8408 spectral pairs in approximately 6.17 s on an RTX 4090, compared with 3.51 s for its CNN baseline, while increasing mean classification accuracy from 95.9% to 99.2% [77]. In contrast, SpectraTr required approximately 73 s of training on the mixed-drug dataset, compared with 48 s for CNN, with closely comparable accuracies at an 80:20 split (99.52% and 99.57%, respectively) [45]. These findings indicate that additional computational cost does not always translate into improved predictive performance, supporting the need to compare Transformers with optimized CNN and chemometric baselines. Assess training cost and inference efficiency separately under clearly specified hardware and workload conditions, as timings across studies are not directly comparable.

Self-supervised approaches such as masked spectral modeling and RamanFormerSSL may reduce dependence on labeled data, while chemistry-informed encoding and constrained prediction can improve the analytical relevance of future models [101,102,103]. Extending self-supervised pretraining to large and diverse spectral collections offers a route towards spectroscopic foundation models that can be adapted to multiple analytical tasks. The DSCF model provides an early example of this direction, with applications in spectral preprocessing, quantification, and interpretation [104]. Successful adaptation will depend on the diversity of pretraining data and reliable transfer across samples, instruments, and acquisition conditions. Transformer models may be particularly useful when prediction depends on relationships between distant spectral regions or interactions among complementary modalities, especially when sufficient independent training data or suitable pretrained representations are available. Their selection should be supported by demonstrable improvements over optimized simpler models under comparable validation conditions. Overall, future progress should be assessed through external validity, reproducibility, interpretability, uncertainty awareness, and practical analytical value rather than accuracy on a single randomly partitioned dataset.

## 5. Conclusions

Transformer models have expanded the analytical capabilities of vibrational spectroscopy by enabling global modeling of spectral sequences and flexible integration with convolutional, generative, and multimodal learning frameworks. Recent studies have applied these models to spectral preprocessing, classification, quantitative analysis, spectra-to-structure prediction, and multimodal data fusion across Raman, IR, NIR, and SERS applications.

The reviewed evidence indicates that Transformers can effectively capture relationships between distant spectral regions and support end-to-end spectral analysis. However, they do not consistently outperform optimized chemometric or CNN-based methods, particularly with small or highly structured datasets. Their practical value therefore depends on the analytical problem, dataset quality, validation strategy, and computational requirements. Future progress will require larger, more representative datasets; realistic external validation; improved interpretability; uncertainty estimation; and greater integration of spectroscopic and chemical knowledge. Transformer models may become valuable components of reliable and transferable workflows for vibrational spectroscopy.

## Figures and Tables

**Table 1:** Summary of the reviewed studies on vibrational spectral classification and component identification.

Authors/Year	Sample Type	Modality	Model	Number of Subjects/Samples	Number of Spectra	Number of Classes	Validation Strategy	Performance/Comparison
Fu et al. 2022 [45]	Drug products	NIR	SpectraTr	333 cefixime + 716 phenytoin products	333 cefixime; 1049 mixed drugs	7/18	80:20 train/test (spectra) ^a^	Accuracy: 100%/99.52%; mixed-drug CNN: 99.57%
Liu et al. 2023 [51]	Cultured bacteria	Raman	Transformer–MLP	8 strains; culture count NR	≥500/strain	8	5-fold CV (spectra); inner 80:20 train/validation	Accuracy: 97.3% (AlexNet: 96.5%)
Tseng et al. 2023 [52]	Clinical blood cultures	SERS	ViT	158 blood cultures	11,774	8 species; 2 Gram types	9464/1161/1149 spectra;	Species: 97.56% (CNN: 92.60%); Gram: 99.30%
Chang et al. 2024 [53]	Murine cell cultures	Raman	RaT	4 samples: 2 B16F10, 2 C2C12	6547	2	5237 train/1310 test (spectra)	Accuracy: 99.69% (CNN: 99.39%)
Weng et al. 2024 [54]	Hair; urine	SERS	TMNet	251 hair donors; 50 urine donors	2495/3930	4 each	30% donor-held-out test; remainder 70:30	Accuracy: hair 99.07%; urine 97.12%
Chen et al. 2024 [55]	Reference compounds	FTIR	Transferable ViT	3753 compounds	5–10/class; 50/class augmented	3753	1 original/class for test; remaining 80:20 after augmentation	Top-1: 84.0% (without transfer: 72.5%)
Yu et al. 2025 [56]	Human tissue	Raman	1D Transformer	90 tissue samples from 90 patients	360	3	10-fold rotating 70:20:10 (spectra)	Accuracy: 93.30 ± 2.53% (PLS–DenseNet: 90.79 ± 3.94%)
Yu et al. 2024 [57]	Breast and lung cell cultures	Raman	Fragment-Fusion Transformer	10 cell lines; cell count NR	≈2300 ^b^	10	80:20 + 5-fold tuning; batch holdout + 4-fold tuning	Random: 94.5% (CNN: 90.1%); batch holdout: 93.4%
Hou et al. 2026 [58]	Human serum	Raman	CET	541 serum samples	541 means (5 scans/sample)	3	20% sample-held-out test; remainder 85:15	Accuracy: 98.2% (SVM: 93.6%)
Wang et al. 2026 [47]	Bacterial colonies	Raman	Ra-ST (Swin)	3 culture batches per strain	7301	14	Leave-one-batch-out CV; 2 additional strains	CV accuracy: 98.2% (RaT: 97.1%)
Meng et al. 2026 [59]	Bacteria and reference materials	Raman	Spectral Transformer	NR	≈2600/class after augmentation	36	70:15:15 (spectra/files); before augmentation	Accuracy at 15 dB: 80.6 ± 0.3% (1D-CNN: 72.9 ± 0.3%)
Huang et al. 2025 [60]	Fruit purees; apple leaves	FTIR; NIR	ACT	NR	Puree: 983; leaves: 5490	2/20	Train/validation/test: 337/329/317; 2500/1250/1740	Accuracy: 96.5 ± 0.4%/79.3 ± 1.4% (AggMapNet/TeaNet: 96.2 ± 0.2%/78.5 ± 4.6%)
O’Leary et al. 2026 [61]	Prostate tissue cores	FTIR imaging	Block-ViT	100 patients; 228 cores	627,171 annotated pixel spectra	6	60:20:20 (patients); 10 repeats	Accuracy: 91.07 ± 3.71%; macro-F1: 90.56 ± 2.36% (CNN macro-F1: 90.23 ± 3.14%)
Procópio et al. 2026 [62]	Human saliva	ATR-FTIR	BSF	65 saliva samples	65	2	Stratified 10-fold CV (samples)	Accuracy: 70 ± 15%; BA: 67 ± 15% (LightGBM BA: 75 ± 18%)
Li et al. 2023 [63]	Analyte solutions on SERS substrates	SERS maps	Attention-based ViT	30/30/25 maps	≈1800–2000/map	2/analyte	Leave-one-map-out CV; separate tuning maps	Detection accuracy: 100%/100%/96%
Zou et al. 2025 [64]	Cultured bacteria	SERS	LSGAN–Transformer	NR	160 original; 2160 after augmentation	4	80:20 train/validation; augmentation order NR	Accuracy: 99.69% (ResNet: 98.15%)
Xue et al. 2026 [65]	Bacterial cultures	Raman	WGAN–Transformer	3 culture replicates/strain	≈300 development + 100 test/strain	8	Separate culture replicates; 5-fold tuning CV	Test: 97.5%; CV: 97.44% (WGAN–ResNet: 88.42%)
Huang & Feng 2025 [66]	Gray/white matter; vessels	Raman	WGAN + CNN–Transformer	NR	654 original; 6000 generated	3	70:15:15 (generated spectra)	Class-wise accuracy: 98.2–99.2% (generated spectra)
Cao et al. 2025 [67]	Bacterial cultures	SERS	Classification–reconstruction Transformer	4 culture batches/species	9600	3 known + 1 held-out species	Batch holdout (3:1); 5-fold tuning CV	Open-set accuracy: 92.5–97.5% (Mahalanobis rejection: 81.1–87.2%)
Mohammad Jafari & Bocklitz 2026 [68]	Single bacterial cells	Raman	Replicate-aware Transformer	9 measurement replicates	5417	6	Nested leave-one-replicate-out CV	Higher replicate sensitivity than chemometric baselines (*p* < 0.05)
McCane et al. 2026 [69]	Glioblastoma and immune cells	Raman	1D-ViT benchmark	3 cell lines; 3 healthy donors	2373	3	5-fold CV; 10 × 80:20 splits (spectra)	1D-CNN outperformed ViT (*p* < 0.01); ≈10× faster
Sineesh & Kamsali 2026 [70]	Minerals; bacterial; chemicals	Raman	Transformer; RamanFormer	30 bacterial isolates; others NR	MLROD: 125,244; Bacteria-ID: 66,000; API: 3510	15/30/32	Held-out acquisition/culture/spectra; 5 runs	Bacteria-ID accuracy: 83.27 ± 0.62%/84.63 ± 0.58% (CNN: 85.89 ± 0.12%)
Chen et al. 2024 [71]	Barley; chickpea; sorghum	NIR	Spectraformer	1200/950/500 grain samples	1200/950/500	24/19/10	70:30 holdout; separate 5-fold CV	CV accuracy: 84.7%/94.7%/84.7% (SG + min–max)
Yu et al. 2025 [72]	PAH standards and mixtures	Raman; SERS	SpecRecFormer	4 reference PAHs; mixture sets	400 reference; 6400 synthetic train; 4850 test	2/target; 4 targets	80:20 before augmentation; 3 experimental test sets	Accuracy: 93.75/89.21/93.63% (LightCNN: 100/68.85/65.69%) ^c^
Wang et al. 2024 [73]	Pure compounds and mixtures	Raman	RST	15 compounds; 60 mixture	4500; 750 pure spectra for training	15 labels	Pure/synthetic training; real-mixture test	Identification: 100% (CNN: 72%); recall: 99.3%
Li et al. 2025 [74]	Polymer particles	FTIR	CNN–Transformer	NR	3521	17	80:20 train/validation (spectra); 50 repeats	Mean validation accuracy: 95.5 ± 0.6%
Patel & Meng 2026 [75]	Cultured isolates	Raman	Two-stage CNN–Transformer	30 isolates	60,000 reference; 3000 fine-tuning; 3000 test	30	Culture-independent test; 5 seeds	Accuracy: 85.0 ± 0.3%; weighted F1: 85.0 ± 0.4%
ChuDuc & Nguyen 2025 [76]	Human skin tissue	Raman	1D-CNN–Transformer	NR	1200	4	10-fold CV; patient grouping NR	Accuracy: 96.8 ± 0.9% (CNN: 92.8 ± 1.5%)
Tan et al. 2025 [77]	Reference compounds; real mixtures	Mid-IR	DeepMIR	10,503 library compounds;	10,503 reference; 84,024 pairs	2	80:10:10 before augmentation; external ATR-FTIR tests	Synthetic-test accuracy: 99.2% (CNN: 95.9%)
Phuong et al. 2025 [78]	Apples; broccoli; leeks; mushrooms	NIR	SMART-NIR	1028 food samples	1028	4	Stratified 5-fold CV (spectra)	Accuracy with noise: 99.01 ± 0.36% (Transformer: 91.29 ± 0.52%)

Abbreviations: NR, not reported; IR, infrared; Mid-IR, mid-infrared; NIR, near-infrared; Vis, visible; FTIR, Fourier-transform infrared; ATR, attenuated total reflection; SERS, surface-enhanced Raman spectroscopy; CV, cross-validation; CNN, convolutional neural network; MLP, multilayer perceptron; ViT, vision Transformer; 1D, one-dimensional; TL, transfer learning; PLS, partial least squares; SVM, support vector machine; BA, balanced accuracy; F1, harmonic mean of precision and recall; WGAN, Wasserstein generative adversarial network; LSGAN, least-squares generative adversarial network; SG, Savitzky–Golay; PAH, polycyclic aromatic hydrocarbon; API, active pharmaceutical ingredient; dB, decibel; MLROD, Machine Learning Raman Open Dataset; RaT, Raman Transformer; CET, categorical embedding Transformer; ACT, analytical-chemistry-informed Transformer; BSF, BioSpectralFormer; RST, Raman Spectral Translation. ^a^ Ref. [45] used test loss for early stopping. ^b^ Ref. [57] gives 2265/2275 spectra in different sections. ^c^ Ref. [72]: standard/expanded/SERS tests.

## Data Availability

No new data were created or analyzed in this study. Data sharing is not applicable to this article.

## Supplementary Materials

The following supporting information can be downloaded at https://www.mdpi.com/article/10.3390/molecules31203745/s1. Table S1: Summary of the reviewed studies on vibrational spectral preprocessing; Table S2: Summary of the reviewed studies on regression and quantitative analysis using vibrational spectroscopy; Table S3: Summary of the reviewed studies on multimodal analysis incorporating vibrational spectroscopy; Table S4: Summary of the reviewed studies on spectra-to-structure prediction.

## References

[1] Larkin P. (2017). Infrared and Raman Spectroscopy: Principles and Spectral Interpretation.

[2] Butler H.J., Ashton L., Bird B., Cinque G., Curtis K., Dorney J., Esmonde-White K., Fullwood N.J., Gardner B., Martin-Hirsch P.L. (2016). Using Raman spectroscopy to characterize biological materials. Nat. Protoc..

[3] Deidda R., Sacré P.-Y., Clavaud M., Coïc L., Avohou H., Hubert P., Ziemons E. (2019). Vibrational spectroscopy in analysis of pharmaceuticals: Critical review of innovative portable and handheld NIR and Raman spectrophotometers. TrAC Trends Anal. Chem..

[4] Paudel A., Raijada D., Rantanen J. (2015). Raman spectroscopy in pharmaceutical product design. Adv. Drug Deliv. Rev..

[5] Kong K., Kendall C., Stone N., Notingher I. (2015). Raman spectroscopy for medical diagnostics—From in-vitro biofluid assays to in-vivo cancer detection. Adv. Drug Deliv. Rev..

[6] Bocklitz T.W., Guo S., Ryabchykov O., Vogler N., Popp J. (2016). Raman based molecular imaging and analytics: A magic bullet for biomedical applications!?. Anal. Chem..

[7] Baker M.J., Hussain S.R., Lovergne L., Untereiner V., Hughes C., Lukaszewski R.A., Thiéfin G., Sockalingum G.D. (2016). Developing and understanding biofluid vibrational spectroscopy: A critical review. Chem. Soc. Rev..

[8] Hands J.R., Dorling K.M., Abel P., Ashton K.M., Brodbelt A., Davis C., Dawson T., Jenkinson M.D., Lea R.W., Walker C. (2014). Attenuated total reflection Fourier transform infrared (ATR-FTIR) spectral discrimination of brain tumour severity from serum samples. J. Biophotonics.

[9] Bokobza L. (2019). Some applications of vibrational spectroscopy for the analysis of polymers and polymer composites. Polymers.

[10] Nazari G., Arami-niya A. (2025). Raman and tip-enhanced Raman spectroscopy studies of polymer blends: A review. J. Polym. Res..

[11] Cozzolino D. (2022). An overview of the successful application of vibrational spectroscopy techniques to quantify nutraceuticals in fruits and plants. Foods.

[12] Mohammadi S., Gowen A., O’Donnell C. (2024). Vibrational spectroscopy data fusion for enhanced classification of different milk types. Heliyon.

[13] de Oliveira Penido C.A.F., Pacheco M.T.T., Lednev I.K., Silveira L. (2016). Raman spectroscopy in forensic analysis: Identification of cocaine and other illegal drugs of abuse. J. Raman Spectrosc..

[14] Almaviva S., Artuso F., Giardina I., Lai A., Pasquo A. (2022). Fast detection of different water contaminants by Raman spectroscopy and surface-enhanced Raman spectroscopy. Sensors.

[15] Armenta S., de la Guardia M. (2014). Vibrational spectroscopy in soil and sediment analysis. Trends Environ. Anal. Chem..

[16] Peris-Díaz M.D., Krężel A. (2021). A guide to good practice in chemometric methods for vibrational spectroscopy, electrochemistry, and hyphenated mass spectrometry. TrAC Trends Anal. Chem..

[17] Ryabchykov O., Guo S., Bocklitz T. (2019). Analyzing Raman spectroscopic data. Phys. Sci. Rev..

[18] Wang D., Zhang M., Zhang Z., Li J., Gao H., Zhang F., Chen X. (2019). Machine learning-based multifunctional optical spectrum analysis technique. IEEE Access.

[19] Mohammad Jafari J., Bocklitz T. (2025). Exploring feature extraction methods for Raman spectroscopy: A comparative study. Anal. Chim. Acta.

[20] Coccato A., Caggiani M.C. (2024). An overview of Principal Components Analysis approaches in Raman studies of cultural heritage materials. J. Raman Spectrosc..

[21] Guo S., Rösch P., Popp J., Bocklitz T. (2020). Modified PCA and PLS: Towards a better classification in Raman spectroscopy-based biological applications. J. Chemom..

[22] Lasalvia M., Capozzi V., Perna G. (2022). A comparison of PCA-LDA and PLS-DA techniques for classification of vibrational spectra. Appl. Sci..

[23] Kyriakides A., Kastanos E., Pitris C. Classification of Raman spectra using support vector machines. Proceedings of the 2009 9th International Conference on Information Technology and Applications in Biomedicine.

[24] Qi Y., Hu D., Jiang Y., Wu Z., Zheng M., Chen E.X., Liang Y., Sadi M.A., Zhang K., Chen Y.P. (2023). Recent progresses in machine learning assisted Raman spectroscopy. Adv. Opt. Mater..

[25] Cui X., Zhao Z., Zhang G., Chen S., Zhao Y., Lu J. (2018). Analysis and classification of kidney stones based on Raman spectroscopy. Biomed. Opt. Express.

[26] Ullah R., Parveen K., Rehan I., Khan S. (2025). Enhancing lung cancer diagnostics through Raman spectroscopy and machine learning. Phys. Scr..

[27] Rinnan Å., van den Berg F.W.J., Engelsen S.B. (2009). Review of the most common pre-processing techniques for near-infrared spectra. TrAC Trends Anal. Chem..

[28] Liu J., Osadchy M., Ashton L., Foster M., Solomon C.J., Gibson S.J. (2017). Deep convolutional neural networks for Raman spectrum recognition: A unified solution. Analyst.

[29] Gerretzen J., Szymańska E., Jansen J.J., Bart J., van Manen H.-J., van den Heuvel E.R., Buydens L.M.C. (2015). Simple and effective way for data preprocessing selection based on design of experiments. Anal. Chem..

[30] Workman J.J. (2018). A review of calibration transfer practices and instrument differences in spectroscopy. Appl. Spectrosc..

[31] Luo R., Popp J., Bocklitz T. (2022). Deep learning for Raman spectroscopy: A review. Analytica.

[32] Acquarelli J., van Laarhoven T., Gerretzen J., Tran T.N., Buydens L.M.C., Marchiori E. (2017). Convolutional neural networks for vibrational spectroscopic data analysis. Anal. Chim. Acta.

[33] Meza Ramirez C.A., Greenop M., Ashton L., Rehman I.U. (2021). Applications of machine learning in spectroscopy. Appl. Spectrosc. Rev..

[34] Walsh J., Neupane A., Koirala A., Li M., Anderson N. (2023). Review: The evolution of chemometrics coupled with near infrared spectroscopy for fruit quality evaluation. II. The rise of convolutional neural networks. J. Near Infrared Spectrosc..

[35] Yang J., Xu J., Zhang X., Wu C., Lin T., Ying Y. (2019). Deep learning for vibrational spectral analysis: Recent progress and a practical guide. Anal. Chim. Acta.

[36] Mishra P., Passos D., Marini F., Xu J., Amigo J.M., Gowen A.A., Jansen J.J., Biancolillo A., Roger J.M., Rutledge D.N. (2022). Deep learning for near-infrared spectral data modelling: Hypes and benefits. TrAC Trends Anal. Chem..

[37] Vaswani A., Shazeer N., Parmar N., Uszkoreit J., Jones L., Gomez A.N., Kaiser Ł., Polosukhin I. Attention is all you need. Proceedings of the 31st International Conference on Neural Information Processing Systems (NIPS 2017).

[38] Xue X., Sun H., Yang M., Liu X., Hu H.-Y., Deng Y., Wang X. (2023). Advances in the application of artificial intelligence-based spectral data interpretation: A perspective. Anal. Chem..

[39] Wu W., Leonardis A., Jiao J., Jiang J., Chen L. (2025). Transformer-based models for predicting molecular structures from infrared spectra using patch-based self-attention. J. Phys. Chem. A.

[40] Ahmad M., Distefano S., Khan A.M., Mazzara M., Li C., Li H., Aryal J., Ding Y., Vivone G., Hong D. (2025). A comprehensive survey for hyperspectral image classification: The evolution from conventional to Transformers and Mamba models. Neurocomputing.

[41] Luong K.-D., Singh A. (2024). Application of Transformers in cheminformatics. J. Chem. Inf. Model..

[42] Cai Y., Lin Y., Cai H., Ni H. (2025). Deep learning in vibrational spectroscopy: Benefits, limitations, and recent progress. J. Chin. Chem. Soc..

[43] He K., Zhang X., Ren S., Sun J. Deep residual learning for image recognition. Proceedings of the 2016 IEEE Conference on Computer Vision and Pattern Recognition (CVPR).

[44] Ba J.L., Kiros J.R., Hinton G.E. (2016). Layer normalization. arXiv.

[45] Fu P., Wen Y., Zhang Y., Li L., Feng Y., Yin L., Yang H. (2022). SpectraTr: A novel deep learning model for qualitative analysis of drug spectroscopy based on transformer structure. J. Innov. Opt. Health Sci..

[46] Gao L., Yan X., Deng L., Xu G., Zhu H. (2024). SST: Sparse self-attention Transformer for infrared spectrum deconvolution. Infrared Phys. Technol..

[47] Wang Y., Xu L., Shang L., Peng H., Liu K., Bao X., Tang X., Liang P., Wang Y., Zheng M. (2026). Deep learning-assisted Raman spectroscopy for rapid lactic acid bacteria identification at the colony level. Spectrochim. Acta A Mol. Biomol. Spectrosc..

[48] Zhao J., Woznicki T., Kusnierek K. (2025). Estimating baselines of Raman spectra based on Transformer and manually annotated data. Spectrochim. Acta A Mol. Biomol. Spectrosc..

[49] Gao L., Yan X., Zhu H. Infrared spectral deconvolution algorithm based on masked pre-training Transformer. Proceedings of the International Conference on Computer Vision and Deep Learning (CVDL 2024).

[50] Gao L., Cui L., Chen S., Deng L., Wang X., Yan X., Zhu H. (2025). AMTrans: Auto-correlation multi-head attention Transformer for infrared spectral deconvolution. Tsinghua Sci. Technol..

[51] Liu B., Liu K., Qi X., Zhang W., Li B. (2023). Classification of deep-sea cold seep bacteria by Transformer combined with Raman spectroscopy. Sci. Rep..

[52] Tseng Y.-M., Chen K.-L., Chao P.-H., Han Y.-Y., Huang N.-T. (2023). Deep learning-assisted surface-enhanced Raman scattering for rapid bacterial identification. ACS Appl. Mater. Interfaces.

[53] Chang M., He C., Du Y., Qiu Y., Wang L., Chen H. (2024). RaT: Raman Transformer for highly accurate melanoma detection with critical features visualization. Spectrochim. Acta A Mol. Biomol. Spectrosc..

[54] Weng S., Wang C., Zhu R., Wu Y., Yang R., Zheng L., Li P., Zhao J., Zheng S. (2024). Identification of surface-enhanced Raman spectroscopy using hybrid Transformer network. Spectrochim. Acta A Mol. Biomol. Spectrosc..

[55] Chen Z., Xie Y., Wu Y., Lin Y., Tomiya S., Lin J. (2024). An interpretable and transferrable Vision Transformer model for rapid materials spectra classification. Digit. Discov..

[56] Yu X.-Y., Chen J., Li L.-Y., Chen F.-E., He Q. (2025). Rapid pathologic grading-based diagnosis of esophageal squamous cell carcinoma via Raman spectroscopy and a deep learning algorithm. World J. Gastroenterol..

[57] Yu Q., Shen X., Yi L., Liang M., Li G., Guan Z., Wu X., Castel H., Hu B., Yin P. (2024). Fragment-Fusion Transformer: Deep learning-based discretization method for continuous single-cell Raman spectral analysis. ACS Sens..

[58] Hou C., Xie F., Wu G., Yu W., Yang H., Yang L., Long X., Yin L., Wang S. (2026). A classification model for early detection of breast cancer by Raman spectroscopy based on categorical embedding Transformer. Chemom. Intell. Lab. Syst..

[59] Meng Y., Christensen J.B., Thirstrup C., Rute L.R., Stergiou K., Komisar D., Ilchenko O., Tornby D.R., Andersen T.E., Aslan H. (2026). Interpretable spectral Transformer for Raman-based bacterial identification across species and strains. Chemosensors.

[60] Huang S., Jin Y., Jin W., Mu Y. (2025). Analytical-Chemistry-Informed Transformer for infrared spectra modeling. Proc. AAAI Conf. Artif. Intell..

[61] O’Leary L., Ferguson D., Hart C., Brown M., Oliveira P., Clarke N., Sachdeva A., Gardner P., Yin H. (2026). Spatial-spectral deep learning for prostate cancer tissue classification in infrared spectroscopy. Anal. Chem..

[62] Procópio L.S., da Silva R.S., Carneiro M.G., Souza P.D. BioSpectralFormer: A Transformer-based architecture for FTIR spectra classification in oral cancer diagnosis. Proceedings of the XXVI Simpósio Brasileiro de Computação Aplicada à Saúde (SBCAS 2026).

[63] Li B., Zappalá G., Dumont E., Boisen A., Rindzevicius T., Schmidt M.N., Alstrøm T.S. (2023). Nitroaromatic explosives’ detection and quantification using an attention-based Transformer on surface-enhanced Raman spectroscopy maps. Analyst.

[64] Zou Y., Li Y., Zhang F., Ge Y., Wang W., Chen M. (2025). Rapid identification of *Litopenaeus vannamei* pathogenic bacteria: A combined approach using surface-enhanced Raman spectroscopy (SERS) and deep learning. Anal. Bioanal. Chem..

[65] Xue Y., Sun B., Zhang Y., Liu M. (2026). An integrated Wasserstein GAN–Transformer deep learning framework for accurate Raman spectral identification of *Escherichia coli* strains. Anal. Chem..

[66] Huang H., Feng Y. Research on Raman spectroscopy-based brain tissue classification using WGAN and Transformer. Proceedings of the 2025 6th International Symposium on Artificial Intelligence for Medical Sciences (ISAIMS 2025).

[67] Cao H., Cheng J., Ma X., Liu S., Guo J., Li D. (2025). Deep learning enabled open-set bacteria recognition using surface-enhanced Raman spectroscopy. Biosens. Bioelectron..

[68] Mohammad Jafari J., Bocklitz T. (2026). Transformer-based classification of bacterial Raman spectra with LOOCV. arXiv.

[69] McCane B., Rijwan I., Fraser-Miller S.J., Neumann S., Maciaczyk D., Maciaczyk J., Gordon K. (2026). Machine learning for Raman spectroscopy glioblastoma classification. J. Chemom..

[70] Sineesh A., Kamsali A.R. (2026). Benchmarking deep learning models for Raman spectroscopy across open-source datasets. Digit. Discov..

[71] Chen Z., Zhou R., Ren P. (2024). Spectraformer: Deep learning model for grain spectral qualitative analysis based on Transformer structure. RSC Adv..

[72] Yu X., Liu T., Kong L., Lan T., Sun Q., Qu F., Liu M., Chen J., Huang M. (2025). SpecRecFormer: Deep learning-driven adaptive component identification of PAH mixtures based on single-component Raman spectra. Anal. Chem..

[73] Wang Z., Li Y., Zhai J., Yang S., Sun B., Liang P. (2024). Deep learning-based Raman spectroscopy qualitative analysis algorithm: A convolutional neural network and Transformer approach. Talanta.

[74] Li F., Tian J., Guo X., Xu L. (2025). Hybrid deep learning framework for environmental microplastic classification: Integrating CNN-based spectral feature extraction and Transformer models. Environ. Pollut..

[75] Patel A., Meng H. (2026). Rapid identification of pathogenic bacteria from Raman spectra with a CNN-Transformer hybrid architecture. J. Data Sci. Intell. Syst..

[76] ChuDuc H., Nguyen T.T. A hybrid 1D-CNN and Transformer architecture for differentiating malignant melanoma from non-melanoma skin cancers using Raman spectroscopy. Proceedings of the 2025 International Conference on Advanced Technologies for Communications (ATC).

[77] Tan L., Wang Y., Zhang H., Sun J., Yang Q., Yang X., Zhang Z., Lu H. (2025). DeepMIR: A hybrid convolutional neural network–Transformer framework for accurate identification of target components from mid-infrared spectra of mixtures. Anal. Chem..

[78] Phuong N.T.H., Nhat P.M., Hieu N.V. (2025). SMART-NIR: A multi-kernel Vision Transformer with Kolmogorov-Arnold Network classifier for near-infrared spectral classification. Informatica.

[79] Guo S., Bocklitz T., Neugebauer U., Popp J. (2017). Common mistakes in cross-validating classification models. Anal. Methods.

[80] Yuan Y., Sun Y., Du F., He L., Peng X., Qu J. (2026). Precisely Identifying Growth Phases of Living Bacteria using Open-Set Deep Learning-Driven Single-Cell Raman Spectroscopy. Anal. Chem..

[81] Jin X., Zhou J., Rao Y., Zhang X., Zhang W., Ba W., Zhou X., Zhang T. (2023). An innovative approach for integrating two-dimensional conversion of Vis–NIR spectra with the Swin Transformer model to leverage deep learning for predicting soil properties. Geoderma.

[82] Koyun O.C., Keser R.K., Şahin S.O., Bulut D., Yorulmaz M., Yücesoy V., Töreyin B.U. (2024). RamanFormer: A Transformer-based quantification approach for Raman mixture components. ACS Omega.

[83] Singh N., Kaur S., Mithraa T., Verma V.K., Kumar A., Choudhary V., Bhardwaj R. (2024). ProTformer: Transformer-based model for superior prediction of protein content in lablab bean (*Lablab purpureus* L.) using Near-Infrared Reflectance spectroscopy. Food Res. Int..

[84] Tian W., Zang L., Li Y., Peng C., Zhang J., Shao H., Xian R., Sun R., Liu F., Tan H. (2025). A Transformer-based framework with generative spectral augmentation for online monitoring of hyaluronic acid fermentation. Carbohydr. Polym..

[85] Liu J., Liu X., Wang X., Lim Z.H., Liu H., Zhao Y., Yu W., Yu T., Hu B. (2025). Rapid COD sensing in complex surface water using Physicochemical-Informed Spectral Transformer with UV–Vis–SWNIR spectroscopy. Environ. Sci. Technol..

[86] Yu Y., Qiao Y., Fan C., Dong M., Cao K. (2025). Machine learning and near-infrared fusion-driven quantitative characterization and detection of protein content in maize kernels. Front. Nutr..

[87] Guo W., Gao S., Ding Y., Dong D. (2025). Simultaneous prediction of multiple soil components using Mid-Infrared Spectroscopy and the GADF-Swin Transformer model. Comput. Electron. Agric..

[88] Yue J., Zhang S., Feng T., Gao L., Nie L., Zang H. (2025). Feasibility study of miniature near-infrared spectroscopy with Transformer for non-destructive detection of *Angelica sinensis*. Microchem. J..

[89] Li J., Wang H., Zhang H., Jiang T. (2025). Multi-Path Attention Fusion Transformer for spectral learning in corn quality assessment. Foods.

[90] Wu T., Li L., Zhu L., Bai W., Lin L., Liu L., Yang L. (2025). Nondestructive detection of thiophanate-methyl pesticide content in cherry tomato based on handheld spectrometer and SpecTransformer algorithm. J. Food Meas. Charact..

[91] Zhou X., Chen C., Lv X., Zuo E., Li M., Wu L., Chen X., Wu X., Chen C. (2024). CMACF: Transformer-based cross-modal attention cross-fusion model for systemic lupus erythematosus diagnosis combining Raman spectroscopy, FTIR spectroscopy, and metabolomics. Inf. Process. Manag..

[92] Li Y., Ji S., Ma L., Shen Y., Yuan G., Bian J., Liu B., Meng F., He N., Wang C. (2025). Elastic scattering spectrum fused with Raman spectrum for rapid classification of colorectal cancer tissues. Anal. Methods.

[93] Xu W., Fu Z., Tang B., Xu J., Li G. (2025). A chemometric approach for FTIR–Raman–LIBS tri-modal spectral fusion: Transformer-based accurate identification of recyclable polymers. Anal. Chim. Acta.

[94] Wu S., Ji S., Yuan M., Jiang J., Luo Y., Hu W. (2025). Multimodal machine learning for molecular identification based on IR, Raman, and NMR spectra. J. Phys. Chem. Lett..

[95] Li J., Shi T., Chen C., Zhou X., Liu W., Lv X., Gao F., Chen C. (2026). A trinity-branch parallel fusion and supervised enhancement network: A multimodal celiac disease diagnosis network based on Transformer and dual-tower supervision. Eng. Appl. Artif. Intell..

[96] Wang H., Ding M., Lan S., Wu T., Du Y. (2026). Interpretable CNN–Transformer multimodal hierarchical fusion network in multivariate calibration. Anal. Chem..

[97] Alberts M., Laino T., Vaucher A.C. (2024). Leveraging infrared spectroscopy for automated structure elucidation. Commun. Chem..

[98] Kanakala G.C., Sridharan B., Priyakumar U.D. (2024). Spectra to structure: Contrastive learning framework for library ranking and generating molecular structures for infrared spectra. Digit. Discov..

[99] Alberts M., Zipoli F., Laino T. (2025). Setting new benchmarks in AI-driven infrared structure elucidation. Digit. Discov..

[100] Zhang C., Ha Y. (2026). Toward complete molecular structure prediction from infrared spectroscopy using deep learning. J. Chem. Inf. Model..

[101] Koyun O.C., Keser R.K., Şahin S.O., Bulut D., Yorulmaz M., Yücesoy V., Töreyin B.U. (2026). RamanFormerSSL: A self-supervised learning-based transformer model for Raman mixture component quantification. Neural Comput. Appl..

[102] Ren P., Zhou R.-G., Li Y. (2025). A self-supervised learning method for Raman spectroscopy based on masked autoencoders. Expert Syst. Appl..

[103] Paidi S.K., Maheshwari P. (2025). RamanMAE: Masked autoencoders enable efficient molecular imaging by learning biologically meaningful spectral representations. Anal. Chem..

[104] Xue B., Bi X., Dong Z., Xu Y., Liang M., Fang X., Yuan Y., Wang R., Liu S., Jiao R. (2025). Deep spectral component filtering as a foundation model for spectral analysis demonstrated in metabolic profiling. Nat. Mach. Intell..

