# Supplementary material for: Transformer Models in Vibrational Spectroscopy: A Review
Source: Molecules. 2026 Oct 19;31(20):3745. doi: 10.3390/molecules31203745 (PMC13707786; doi:10.3390/molecules31203745)
Supplement: Supplementary file 1 [file molecules-31-03745-s001.zip › molecules-4590634-supplementary.pdf]

Transformer models in vibrational spectroscopy: A review

Jamile Mohammad Jafari[a,b], Thomas Bocklitz[a,b*]

[a] Leibniz Institute of Photonic Technology, Member of Leibniz Health Technologies, Member of the Leibniz Centre for Photonics in Infection Research (LPI), Albert-Einstein-Strasse 9, 07745, Jena, Germany

[b] Institute of Physical Chemistry (IPC) and Abbe Center of Photonics (ACP), Friedrich Schiller University Jena, Member of the Leibniz Centre for Photonics in Infection Research (LPI), Helmholtzweg 4, 07743, Jena, Germany

*Thomas.bocklitz@uni-jena.de

Table S1. Summary of the reviewed studies on vibrational spectral preprocessing.

Authors / Year	Sample type	Modality	Model	Subjects / samples	Number of spectra	Preprocessing task	Validation strategy	Performance / comparison
Zhao et al. 2025 [48]	Biological materials	Raman	1dTrans	8 materials; specimens NR	56 original; 16,800 augmented[a]; 259 additional	Baseline correction	5-fold CV (augmented); unseen-material tests	MAE: 4.47×10^{-5} (ResUNet: 1.06×10^{-4})
Gao et al. 2024 [49]	Reference spectra	IR	Masked pre-training Transformer	NR	2,000 synthetic training	Deconvolution/ denoising	Split NR	RMSE: 0.0122 (NPL: 0.0125)
Gao et al. 2024 [46]	Reference spectra	IR	SST	NR	2,510 synthetic	Deconvolution/ denoising	2,000 train/510 validation; source grouping NR	RMSE: 0.0123 (NPL: 0.0125)
Gao et al. 2025 [50]	Reference spectra; powder	IR	AMTrans	NR; powder test: 1	4,000 synthetic training	Deconvolution/ denoising	Split NR; qualitative powder test	RMSE: 0.0121 (UPL: 0.0125)

Abbreviations: NR, not reported; IR, infrared; CV, cross-validation; MAE, mean absolute error; RMSE, root mean squared error; SST, sparse self-attention Transformer; AMTrans, auto-correlation multi-head attention Transformer; NPL, unpaired self-supervised learning blind deconvolution network; UPL, unpaired self-supervised learning blind deconvolution network. Model labels follow the cited studies.

[a] 16,800 augmented pairs = 56 originals × 300; source-independent CV grouping was not reported. Refs. [46, 49, 50]: RMSE for synthetic degradation with Gaussian kernel size 6.

Table S2. Summary of the reviewed studies on regression and quantitative analysis using vibrational spectroscopy.

Authors / Year	Sample type	Modality	Model	Subjects / samples	Number of spectra	Prediction target	Validation strategy	Performance / comparison
Jin et al. 2023 [81]	Soil (LUCAS)	Vis–NIR	GADF–Swin Transformer	19,036; sand/clay: 17,939	1/sample	OC, N, CEC, pH, sand, clay	70:30 train/test (samples); 5-fold CV within training	R^2: 0.74–0.95; OC RMSE: 23.25 g/kg (CNN: 24.18)
Koyun et al. 2024 [82]	Chemical mixtures	Raman	RamanFormer	37 + 6 test mixtures	NR	Component fractions	LOOCV + 6-mixture external test	RMSE: 1.6% (ResNet50: 3.2%)
Singh et al. 2024 [83]	Lablab bean seeds	Vis–NIR	ProTformer	112 samples	112	Protein	80:20 train/test (samples); 5-fold tuning CV	R^2: 0.977 (1D-CNN: 0.956)
Tian et al. 2025 [84]	HA fermentati on broth	NIR	WGAN-GP + Transformer	5 batches	480 (96/batch)	HA molecular weight / concentration	3 training/2 test batches; 10-fold tuning CV	RMSEP: 94,136 Da / 230 mg/L (SPA–PLS: 121,876 / 374.6)
Liu et al. 2025 [85]	Spiked/nat ural water	UV–Vis–SWNIR	PIST	98 spiked; 102 natural	200 means (10 scans/sample)	COD	5-fold CV (samples); 50 runs	Natural water RMSE: 0.6874 mg/L (CNN: 0.9734)
Yu et al. 2025 [86]	Maize powder	NIR	Transformer	90 samples	90 means (3 scans/sample)	Protein	SPXY: 60 calibration/30 validation	RMSEP: 0.321% (SPA–PLSR: 0.301%)
Guo et al. 2025 [87]	Soil (KSSL)	IR (FTIR)	Pretrained GADF–Swin Transformer	19,109 retained	19,109	C, CEC, clay, K, N, OC, P, pH, sand	70:30 train/test (samples)[a]	R^2: 0.75–0.98; OC RMSE: 0.71% (CNN: 0.60%)
Yue et al. 2025 [88]	Angelica sinensis	NIR	Multi-task Transformer	120 samples	3 per sample per instrument	Ligustilide/ferulic acid	90 calibration/30 validation; validation-based stopping	MicroNIR R^2: 0.88/0.94 (PLS: 0.95/0.77)
Li et al. 2025 [89]	Corn	NIR	SpecTran	80 samples	80; 2,000 after interpolation	Moisture, starch, oil, protein	Split and augmentation order NR	Mean R^2: 0.483 (CNN: 0.395)
Wu et al. 2025 [90]	Cherry tomatoes	UV–Vis–NIR	SpecTransforme r	300 samples	300 means (3 scans/sample)	Pesticide dilution labels[b]	80:20 train/validation (samples)	R^2: 0.91; RMSEP: 1.05[b]

Abbreviations: NR, not reported; IR, infrared; NIR, near-infrared; Vis, visible; SWNIR, short-wave near-infrared; UV–Vis, ultraviolet–visible; FTIR, Fourier-transform infrared; CV, cross-validation; LOOCV, leave-one-out cross-validation; GADF, Gramian angular difference field; CNN, convolutional neural network; 1D, one-dimensional; WGAN-GP, Wasserstein

generative adversarial network with gradient penalty; PIST, physicochemical-informed spectral Transformer; HA, hyaluronic acid; OC, organic carbon; CEC, cation exchange capacity; COD, chemical oxygen demand; PLS, partial least squares; PLSR, partial least squares regression; SPA, successive projections algorithm; SPXY, sample partitioning by joint X–Y distances; SVR, support vector regression; R^2, coefficient of determination; RMSE, root mean squared error; RMSEP, root mean squared error of prediction; LUCAS, Land Use/Cover Area frame statistical Survey; KSSL, Kellogg Soil Survey Laboratory. C, N, K, and P denote carbon, nitrogen, potassium, and phosphorus, respectively.

[a] Ref. [87] used test data to tune its SVR baseline. [b] Ref. [90]: log-dilution scale, not measured residue concentration.

Table S3. Summary of the reviewed studies on multimodal analysis incorporating vibrational spectroscopy.

Authors / Year	Sample type	Modality	Model	Subjects / samples	Number of spectra	Task / target	Validation strategy	Performance / comparison
Zhou et al. 2024 [91]	Human serum	Raman + IR (FTIR) + metabolomics	CMACF	119 subjects	119 means/modality (triplicates)	HC/SLE/LN (3 classes)	5-fold CV; 80:20 division (nesting NR)	Accuracy: 99.44% (Tn: 95.51%)
Li et al. 2025 [92]	Colorectal tissue	Raman + elastic scattering	Spec-Transformer	20 tissues	2,000/modality; augmentation	Normal/cancer (2 classes)	80:20 train/test; 20% inner validation; grouping NR	Accuracy: 97.00% (ResNet: 96.10%)
Xu et al. 2025 [93]	Polymers	IR (FTIR) + Raman + LIBS	GA-optimized SCT	122 samples	NR	Polymer type (4 classes)	5-fold CV; specimen grouping NR	Best-run accuracy: 99.23% (InceptionNet: 96.80%)
Wu et al. 2025 [94]	Molecular datasets	IR + Raman + $^{13}C/^{1}H$ NMR	TranSpec	QM9: 125,745; SDBS: 1,630	4 per molecule[a]	SMILES generation	80:10:10 (molecules); SDBS evaluation	QM9 Top-1/10: 72.7/92.3% (before ensemble)
Li et al. 2026 [95]	Human serum	Raman + FTIR + metabolomics	TFS-Net	60 subjects (30 CD; 30 HC)	Raman/FTIR: 180 each; triplicates averaged	CD/HC (2 classes)	Subject-level 5-fold CV; non-nested tuning	Accuracy: 95.82 ± 5.05% (TFN: 76.92%)
Wang et al. 2026 [96]	Mangoes	UV–Vis–NIR + metadata	CNN–Transformer	NR	11,691	Dry matter	Random 70:20:10; specimen grouping NR	R^2: 0.939; RMSE: 0.613 (spectral-only RMSE: 1.03)
Wang et al. 2026 [96]	Tobacco	NIR + acquisition date	CNN–Transformer	NR	2,330	Tn, Ts, Trs, Wet	Random 70:20:10; specimen grouping NR	RMSE: 0.0722 / 0.722 / 0.636 / 0.166[b]

Abbreviations: NR, not reported; IR, infrared; FTIR, Fourier-transform infrared; NIR, near-infrared; UV–Vis, ultraviolet–visible; LIBS, laser-induced breakdown spectroscopy; NMR, nuclear magnetic resonance; HC, healthy controls; SLE, systemic lupus erythematosus; LN, lupus nephritis; CD, celiac disease; GA, genetic algorithm; SCT, spectral classification Transformer; CNN, convolutional neural network; TFN, tensor fusion network; CMACF, cross-modal attention cross-fusion; TFS-Net, trinity-branch parallel fusion and supervised enhancement network; CV, cross-validation; SMILES, Simplified Molecular Input Line Entry System; SDBS, Spectral Database for Organic Compounds; R^2, coefficient of determination; RMSE, root mean squared error; Tn, total nicotine; Ts, total sugars; Trs, total reducing sugars; Wet, wet-weight index.

[a] QM9-derived spectra are calculated; SDBS combines measured IR/Raman and calculated NMR, with incomplete modality coverage. [b] Ref. [96]: RMSE values follow the reported target scales; units were not specified.

Table S4. Summary of the reviewed studies on spectra-to-structure prediction.

Authors / Year	Sample type	Modality	Model	Subjects / samples	Number of spectra	Prediction task	Validation strategy	Performance / comparison
Alberts et al. 2024 [97]	Simulated/ experimental	IR	Encoder–decoder Transformer[a]	Small molecules; NIST subset	634,585 simulated; 3,453 experimental	SMILES generation	Simulated 85:5:10; experimental 70:10:20	Experimental Top-1/10: 44.4/69.8%
Kanakala et al. 2024 [98]	Simulated	IR	SMEN	121,944 QM9 molecules	121,944	Library retrieval	Held-out QM9 molecules[c]; 27-molecule NIST transfer test	Full-library Top-1/10: 81.4/99.3%
Kanakala et al. 2024 [98]	Simulated	IR	SMEN–SD	121,944 QM9 molecules	121,944	SMILES generation	Held-out QM9 molecules[c]; 27-molecule NIST transfer test	Greedy: 51%; 3/5 beams: 71/73%
Wu et al. 2025 [39]	Simulated/ experimental	IR	Patch-based Transformer[a]	IBM/QM9S/ NIST	634,585 / 127,468 / 7,178	SMILES generation	Simulated 85:5:10; NIST test: 782; remainder 95:5	NIST Top-1/10: 54.99/78.45%
Alberts et al. 2025 [99]	Simulated/ experimental	IR	Improved patch-based Transformer[a]	Simulations; NIST	1,399,806 simulated; 3,453 experimental[b]	SMILES generation	5-fold CV (experimental fine-tuning)	Top-1/10: 63.79/83.95% (earlier: 44.39/69.79%)
Zhang and Ha 2026 [100]	Simulated	IR	Adapted CoCa	ChEMBL subset	17,116	SMILES generation	80:10:10; 1,710-molecule test set	1/10-attempt accuracy: 16.26/21.75%

Abbreviations: IR, infrared; NIST, National Institute of Standards and Technology; SMILES, Simplified Molecular Input Line Entry System; SMEN, Spectra and Molecule Encoder Network; SD, SMILES decoder; CoCa, Contrastive Captioner; CV, cross-validation.
Retrieval and generation metrics are task-specific. [a] Molecular formula supplied. [b] Ref. [99]: Results/Methods give 3,453/3,455 experimental spectra.
